# Repeat DNA methylation is modulated by adherens junction signaling

**DOI:** 10.1038/s42003-024-05990-4

**Published:** 2024-03-07

**Authors:** Lisa-Marie Brenner, Florian Meyer, Haiqian Yang, Anja R. Köhler, Pavel Bashtrykov, Ming Guo, Albert Jeltsch, Cristiana Lungu, Monilola A. Olayioye

**Affiliations:** 1 Institute of Cell Biology and Immunology, University of Stuttgart, Allmandring 31, 70569 Stuttgart, Germany; 2 Department of Mechanical Engineering, Massachusetts Institute of Technology, Cambridge, 02139 MA USA; 3 Institute of Biochemistry and Technical Biochemistry, University of Stuttgart, Allmandring 31, 70569 Stuttgart, Germany; 4 Stuttgart Research Center Systems Biology (SRCSB), University of Stuttgart, Nobelstraße 15, 70569 Stuttgart, Germany

**Keywords:** Cadherins, Centromeres, Methylation

## Abstract

Through its involvement in gene transcription and heterochromatin formation, DNA methylation regulates how cells interact with their environment. Nevertheless, the extracellular signaling cues that modulate the distribution of this central chromatin modification are largely unclear. DNA methylation is highly abundant at repetitive elements, but its investigation in live cells has been complicated by methodological challenges. Utilizing a CRISPR/dCas9 biosensor that reads DNA methylation of human α-satellite repeats in live cells, we here uncover a signaling pathway linking the chromatin and transcriptional state of repetitive elements to epithelial adherens junction integrity. Specifically, we find that in confluent breast epithelial cell monolayers, α-satellite repeat methylation is reduced by comparison to low density cultures. This is coupled with increased transcriptional activity at repeats. Through comprehensive perturbation experiments, we identify the junctional protein E-cadherin, which links to the actin cytoskeleton, as a central molecular player for signal relay into the nucleus. Furthermore, we find that this pathway is impaired in cancer cells that lack E-cadherin and are not contact-inhibited. This suggests that the molecular connection between cell density and repetitive element methylation could play a role in the maintenance of epithelial tissue homeostasis.

## Abstract (web summary)

By using a CRISPR/dCas9 biosensor that reads DNA methylation of α-satellite repeats in live cells, we identify a signaling pathway linking the chromatin and transcriptional state of repeats to the integrity of epithelial adherens junctions.

## Introduction

DNA methylation is an essential chromatin modification, which is implicated in a variety of cellular processes by regulating gene transcription and heterochromatin formation^1,2^. In mammals, this mark is introduced by DNA methyltransferases (DNMTs) at the fifth position of cytosine bases (5mC), typically in a CpG dinucleotide context. Ten-eleven translocation (TET) enzymes initiate active removal of the modification^3–5^. Although 60–80% of all CpGs are methylated, these sites are not randomly distributed along the sequence of the human genome^3^. CpG dinucleotides cluster within islands, which are present at the promoters of housekeeping and developmental regulated genes and are generally protected from DNA methylation. Methylation of these regions is associated with transcriptional silencing and involves the tight control of DNMT and TET enzyme activities^2,3,5^

Although CpG methylation plays an important role at gene promoters, these functional elements represent only a small fraction of the genome where DNA methylation takes place. In fact, most methylated CpG sites are found within repetitive DNA, which comprises around one half of our genome^2,6^. In general, repetitive DNA sequences are considered to have a high steady-state level of DNA methylation, which is important to prevent repeat DNA recombination and transcription and to maintain proper genome structure^2,6^. Hypomethylation of repeat elements such as the satellite sequences, a class of repeats found on all human centromeres, is frequently observed in cancer^2,6,7^. This has been linked to genome instability and to alterations in the structure of the cell nucleus^2,8^. Of note, although satellite repeats are embedded in a generally repressive chromatin environment, they are still transcriptionally competent^7,9^. While overexpression of centromeric α-satellites induces mitotic errors, a certain degree of satellite DNA transcription is required to establish the function of the centromere^2,7,10,11^. This suggests that the chromatin markup of repetitive DNA is not statically repressive but it is subject to local regulation. This hypothesis is supported by mass spectrometry studies on cell lines treated with the well-characterized DNMT inhibitor 5-deoxy-azacytidine (5-aza-dC). Here, a strong reduction of non-genic DNA methylation was observed after just two hours of treatment. This change was significantly higher than what could be expected based on passive DNA demethylation^3,12^.

Although DNA methylation plays important roles in regulating how cells interact with their environment^5,13,14^, the extracellular signaling cues that trigger remodeling of the DNA methylation landscape have remained largely unclear. In particular for repetitive DNA sequences, this has been complicated by methodological challenges to detect the methylation of these sequences. Long-read methylome sequencing has been recently employed to generate DNA methylation annotations across a complete telomere-to-telomere human genome assembly^9,15^. Although this approach has yielded comprehensive insights into repeat DNA methylation at single base pair resolution, this method is technically demanding and requires cell lysis, resulting in the loss of information about cellular physiology. To overcome these limitations, we here used a CRISPR/dCas9 fluorescence complementation-based sensor, which allows the live visualization of DNA methylation on human satellite repeats^16^. By stably expressing this sensor in the untransformed breast epithelial cell line MCF10A, we identified a new pathway directly linking the methylation of satellite DNA and its transcriptional state to cell density. Perturbation studies identified the adherens junction protein E-cadherin as a key mediator of this signaling cascade. Notably, breast cancer cell lines that do not express E-cadherin and that lack cell contact inhibition, did not respond to cell density changes by altering their repetitive DNA methylation signature. We anticipate that this newly discovered pathway is important to maintain the homeostasis of normal epithelia and its deregulation could promote cancer progression.

## Results

### Establishment of reporter cells to study DNA methylation of α-satellite repeats

**Fig. 1:**
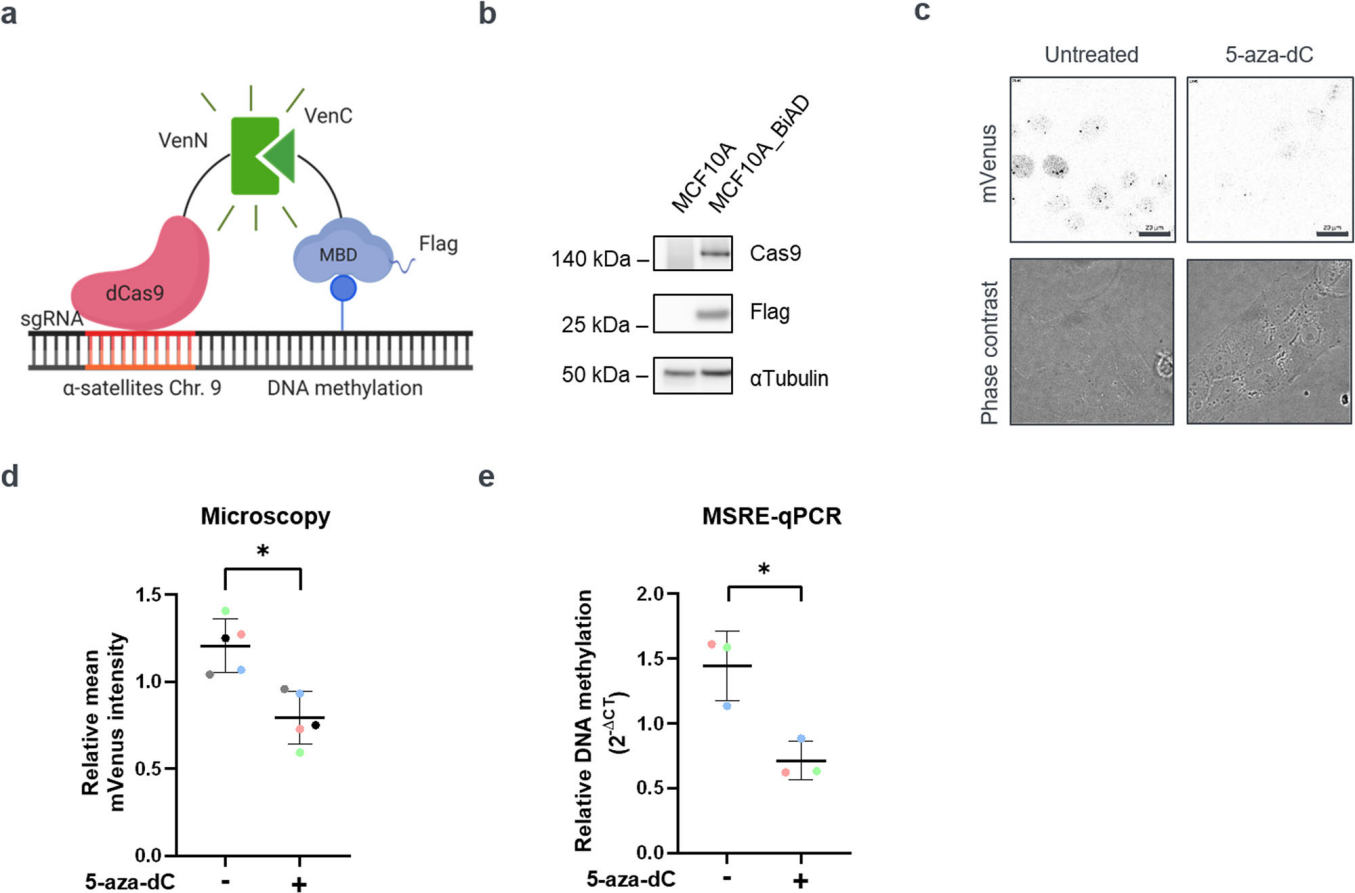
Establishment of a stable MCF10A reporter cell line for DNA methylation readout at α-satellites. **a** Scheme of the BiAD sensor used in this study^16^. Together with the sgRNA, the anchor domain dCas9 (shown in red) recognizes the α-satellite sequences on chromosome 9. The detector domain, the MBD-domain (shown in blue) recognizes the 5mCpG modified sites. Both modules are fused to non-fluorescent fragments of mVenus (VenN and VenC). The occurrence of DNA methylation at the α-satellites leads to binding of the domains in close spatial proximity and reconstitution of a functional mVenus fluorophore. The figure was generated using Biorender. **b** MCF10A cells with stable expression of the BiAD modules (hereafter named MCF10A-BiAD) were generated by lentiviral transduction and the expression of the BiAD modules was validated by Western blot on cell lysates isolated from a clonal population. Antibodies recognizing Cas9 and the Flag-tagged MBD-domain were used. α-Tubulin served as a loading control. Lysates from the parental cells were used as antibody specificity control. Antibody probings were performed on the same membrane, which was cut in horizontal strips accordingly to the expected molecular weight of the target proteins. See also Fig. S8a for the uncropped Western Blot membranes. **c** 5-aza-dC treatment documents the 5mC specificity of the MCF10A-BiAD reporter cells. Shown are representative fluorescence microscopy images and corresponding phase contrast images of the reporter cells. Left panel: control MCF10A-BiAD cells display 2–3 fluorescent spots per nucleus. Right panel: MCF10A-BiAD cells treated for 24 h with 0.25 µM 5-aza-dC display reduced fluorescence intensity at the spots. Both images were acquired and are displayed using the same image settings. Scale bar is 20 µm. **d** Quantification of the relative mean mVenus fluorescence intensity of the immunofluorescence experiments representatively shown in (**c**). *n* = 5, *N* = 80–120. Statistical testing was performed via two-tailed paired *t* test, *p < 0.05. **e** Relative methylation-sensitive restriction digestion coupled with qPCR (MSRE-qPCR) was used to determine the methylation level of α-satellite repeats. Genomic DNA was isolated from MCF10A cells after 24 h treatment with 0.25 µM 5-aza-dC. Mock treated cells were used as control. *n* = 3. Statistical testing was performed using a two-tailed unpaired *t* test *p < 0.05. **d**, **e** In the dot blots, each dot is the mean value obtained for one biological repeat, the line indicates the mean of all biological repeats, and error bars represent their standard deviation. Paired measurements are indicated with the color coding.

To investigate which cellular signals regulate the DNA methylation at heterochromatic repeats, we used a previously described BiAD sensor that detects the DNA methylation at α-satellite repeats by fluorescence microscopy^16^ (Fig. 1a). This fluorescence complementation – based tool consists of an anchor domain, dCas9, and a detector domain, the MBD-domain of MBD1^17,18^, which recognizes 5mC modifications. Both proteins are fused to non-fluorescent fragments of the fluorescent protein mVenus. If DNA methylation is present at the target genomic locus, the domains come in close spatial proximity and the functional fluorophore reconstitutes, leading to a fluorescent signal. The breast epithelial cell line MCF10A was selected as a model system, since it is non-transformed and thus retains normal physiological features and molecular control mechanisms^19^. To facilitate data analysis, a sgRNA was used that targets α-satellite repeat sequences preferentially enriched on chromosome 9^16,20^. Following lentiviral transduction with the BiAD modules, stable cell populations expressing all required parts were generated, which revealed methylation specific nuclear fluorescent foci in line with α-satellite methylation in the MCF10A cell line^21,22^ (Fig. S1a). To achieve a more homogeneous expression of the modules and facilitate quantitative analysis, we next established a clonal subline by fluorescence-activated cell sorting (FACS). The expression of the MBD and dCas9 modules in this reporter cell line, thereafter referred to as MCF10A-BiAD, was validated via Western blot (Fig. 1b). To confirm the methylation specificity of the complementation signal, 5-aza-2′-deoxycytidine (5-aza-dC) was employed, an established inhibitor of the DNA methyltransferases. In line with previous work^16^, a decrease in the complementation signal was observed after 5-aza-dC treatment (Fig. 1c, d). This was confirmed by methylation sensitive restriction enzyme digest coupled with qPCR (MSRE-qPCR) (Fig. S2), which revealed an approximately 50% reduction in DNA methylation at α-satellites following 24 h of drug treatment (Fig. 1e). In sum, this characterization shows that the MCF10A-BiAD reporter cell line can be used to identify changes of DNA methylation at α-satellite repeats.

### The DNA methylation of α-satellite repeats is modulated by cell density

**Fig. 2:**
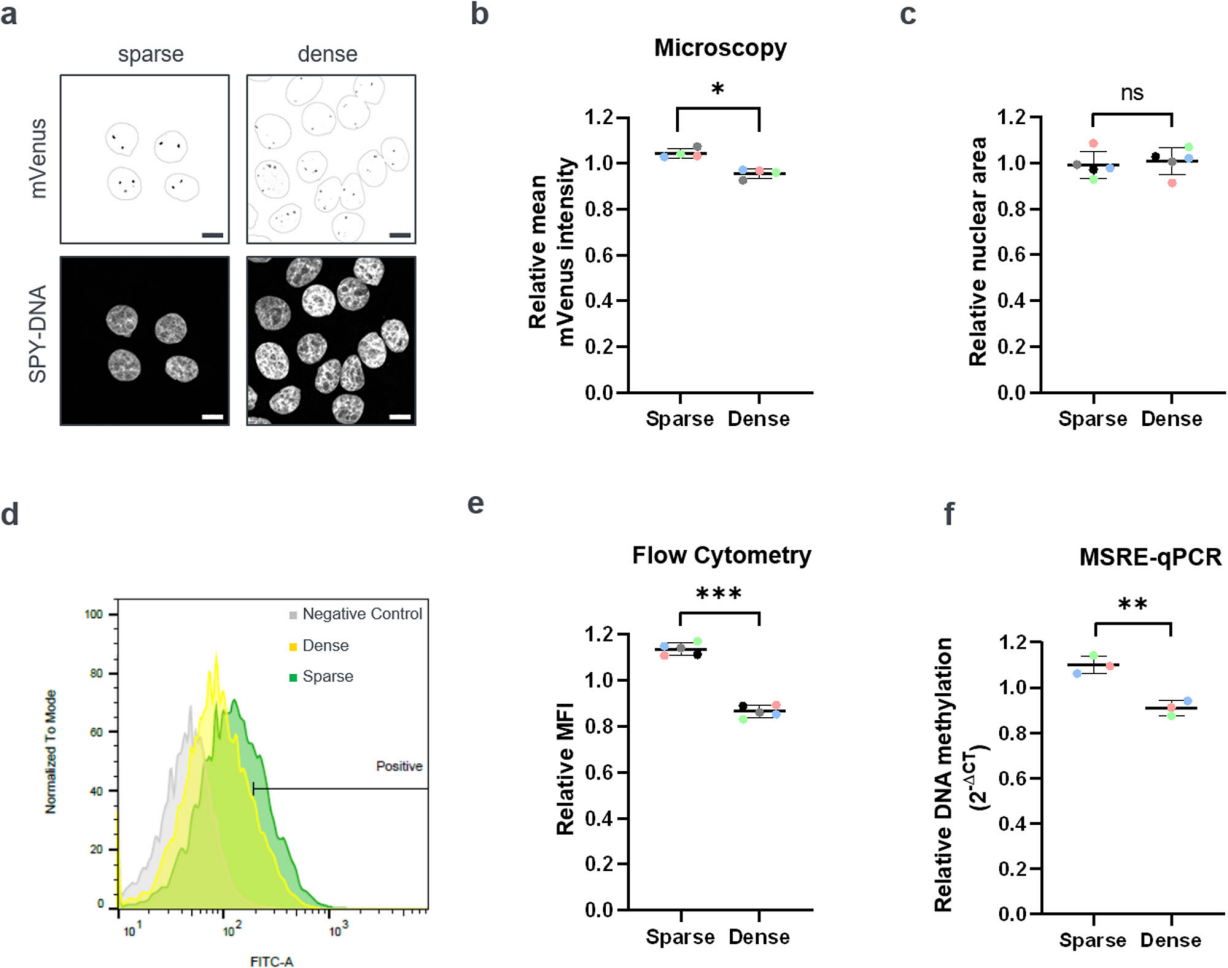
DNA methylation levels of α-satellites are modulated by cell density. **a** MCF10A-BiAD cells were seeded under sparse (5 × 10^3^ cells/cm^2^) or dense (10^5^ cells/cm^2^) conditions and imaged 24 h later. The nuclei were counterstained with SPY650-DNA, which was used to define a nuclear mask (superimposed on the mVenus signal in the top panel). Images were acquired and are displayed using identical settings. Scale bar is 10 µm. **b** Quantification of the immunofluorescence experiments representatively shown in (**a**). *N* = 30–60, *n* = 4. Statistical testing was performed using a two-tailed paired *t* test. *p < 0.05. **c** Quantification of the average nuclear area of MCF10A-BiAD cells grown under sparse and dense conditions. 7 consecutive confocal sections were used for each field of view to generate orthogonal projections of the SPY650-DNA stained nuclei. The average nuclear area was extracted in CellProfiler. *N* = 50–130, *n* = 5. Statistical testing was performed using a two-tailed paired *t* test. ns, not significant. **d** Flow cytometry measurement of the mVenus signal in MCF10A-BiAD reporter cells cultured for 24 h under sparse (green) and dense (yellow) conditions. Parental MCF10A cells were used as a control to gate for the FITC positive population. 10^4^ cells were used for gating for each sample. **e** Quantification of the relative median fluorescence intensity of the flow cytometry experiments representatively shown in (**d**). *n* = 5. Statistical testing was performed using a two-tailed paired *t* test. ***p < 0.001. **f** Relative methylation-sensitive restriction digestion coupled with qPCR (MSRE-qPCR) was used to measure the methylation levels at α-satellite repeats in genomic DNA extracted from MCF10A cells cultured under sparse or dense conditions. *n* = 3. Statistical testing was performed using a two-tailed unpaired *t* test. **p < 0.01. **b**, **c**, **e**, **f** In the dot blots, each dot is the mean value obtained for one biological repeat, the line indicates the mean of all biological repeats, and error bars represent their standard deviation. Paired measurements are indicated with the color coding.

While screening for cellular signals which influence DNA methylation at heterochromatic repeats, we observed that the confluency state of the cells influences the intensity of the mVenus complementation signal. To follow up on this intriguing observation, we performed systematic experiments where the cells were seeded under defined culture densities. 5 × 10^3^ cells/cm^2^ were used for sparse conditions, while 10^5^ cells/cm^2^ were seeded to generate densely packed cultures. Notably, low density cultures showed a significantly brighter mVenus signal than cells cultured at high density (Fig. 2a, b). This was observed already within 24 h after seeding, ruling out nutrient depletion as the cause of lower signal intensities. Furthermore, no change in cell cycle distribution was observed among culture conditions, ruling out cell cycle arrest (Fig. S3a, b). Flow cytometry-based readouts (Fig. 2d, e) confirmed the microscopy quantifications. Importantly, these changes were not due to alterations in the expression levels of the BiAD sensor modules (Fig S1b, c) or mislocalization of the dCas9 domain (Fig. S1d). Furthermore, the nuclear area (Figs. 2c, S1e) was similar regardless of the culturing conditions, indicating that the changes in fluorescence intensity were not caused by severe compression of the nuclei. To validate the changes in DNA methylation through an independent method, MSRE-qPCR was performed on the parental MCF10A cell line. This confirmed the fluorescence-based readouts observed with the reporter, because a significantly lower DNA methylation level was observed at α-satellite sequences in high density cultures than under sparse cultivation conditions (Fig. 2f). In sum, these data demonstrate that in MCF10A cells, the DNA methylation levels of heterochromatic repeats responds to cell culture density.

### E-cadherin connects cell density to the methylation levels of α-satellite repeats

**Fig. 3:**
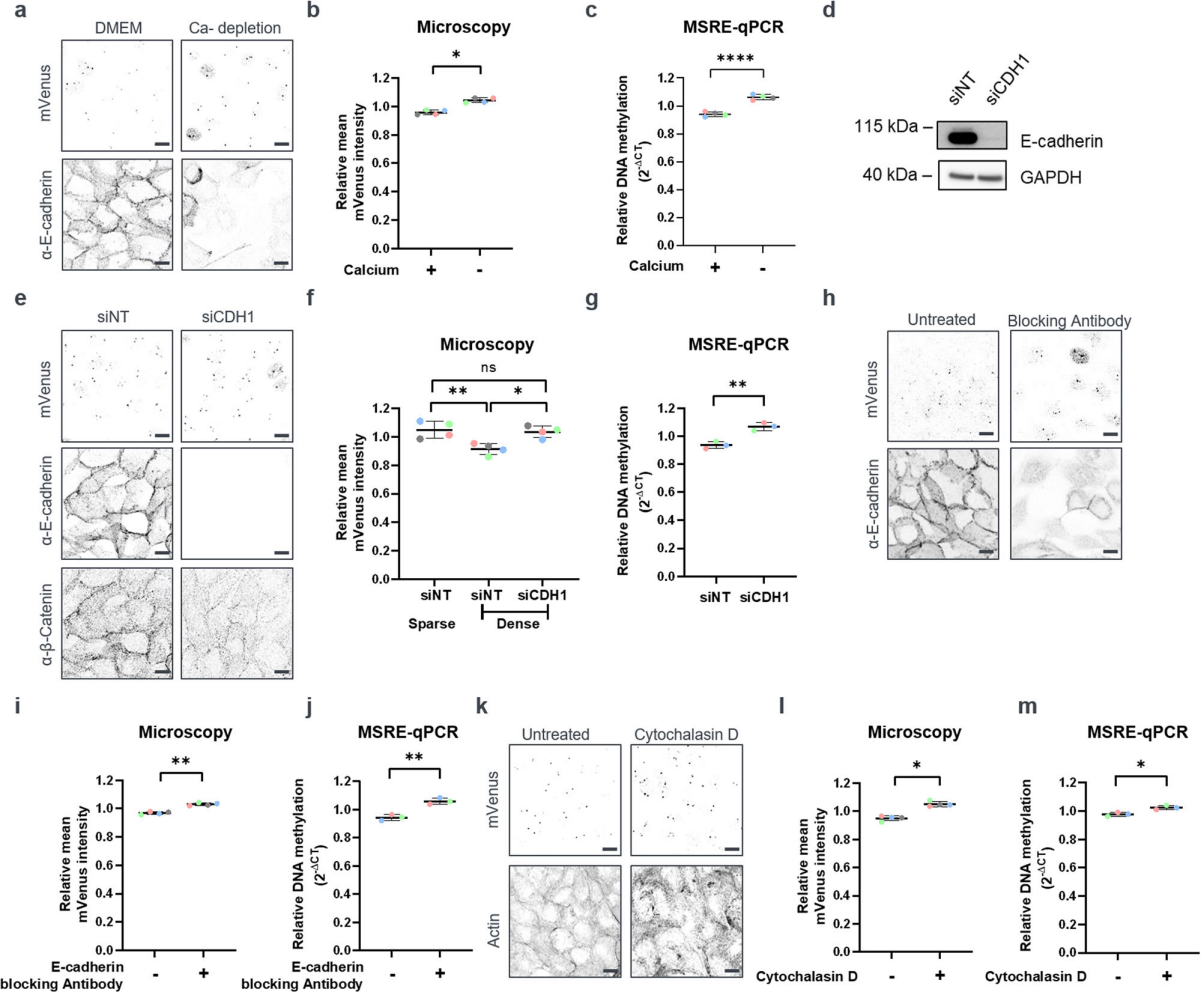
E-cadherin engagement modulates DNA methylation at heterochromatic repeats. **a** Representative fluorescence microscopy images of the MCF10A-BiAD reporter cells grown for 24 h in either complete (left, DMEM) or low calcium medium (right, Ca^2+^ depletion). E-cadherin staining was used to validate the disruption of cell-cell contacts upon Ca^2+^ depletion. Images were acquired and are displayed using identical settings. Scale bar is 10 µm. **b** Quantification of the calcium depletion experiments representatively shown in (**a**) by fluorescence intensity measurements. *N* = 60–100, *n* = 4. Statistical testing was performed using a two-tailed paired *t* test. *p < 0.05. **c** Quantification of the calcium depletion experiments representatively shown in (**a**) by MSRE-qPCR. *n* = 4. Statistical testing was performed using a two-tailed unpaired *t* test. ****p < 0.0001. **d**–**g** MCF10A-BiAD cells were analyzed 72 h post transfection with either control (siNT) or an E-cadherin targeting siRNA (siCDH1). **d** Western blot analysis verifies E-cadherin knock-down. GAPDH was used as a loading control. Antibody probings were performed on the same membrane, which was cut in horizontal strips accordingly to the expected molecular weight of the target proteins. See also Fig. S8b for the uncropped Western Blot membranes. **e** Representative fluorescence microscopy images of dense MCF10A-BiAD cells treated with the indicated siRNAs. E-cadherin staining was used to validate the siRNA knock-down. β-catenin staining was employed for cell shape recognition. Images were acquired and are displayed using identical settings. Scale bar is 10 µm. **f** Quantification of E-cadherin knock-down experiments representatively shown in (**e**) by fluorescence intensity measurements. Sparse cells transfected with siNT siRNA are included as reference. *N* = 20–60, *n* = 4. Statistical testing was performed using a one-way ANOVA. ns, not significant, *p < 0.05, **p < 0.01. **g** Quantification of E-cadherin knock-down experiments representatively shown in (**e**) by MSRE-qPCR. *n* = 3. Statistical testing was performed using a two-tailed unpaired *t* test. ns, not significant, **p < 0.01. **h**–**j** Densely seeded MCF10A-BiAD cells were treated with an E-cadherin blocking antibody and analyzed 24 h later. **h** E-cadherin staining was used to validate the efficient disruption of adherens junctions by the E-cadherin blocking antibody. Shown are representative fluorescence microscopy images that were acquired and are displayed using identical settings. Scale bar is 10 µm. **i** Quantification of the mVenus sensor signal in the E-cadherin blocking experiments representatively shown in (**h**) by fluorescence intensity measurements. *N* = 30–90, *n* = 4. Statistical testing was performed using a two-tailed paired *t* test. **p < 0.01. **j** Quantification of E-cadherin blocking experiments representatively shown in (**h**) by MSRE-qPCR. Genomic DNA was isolated 24 h after the addition of the blocking antibody. *n* = 3. Statistical testing was performed using a two-tailed unpaired *t* test. **p < 0.01. **k**–**m** MCF10-BiAD cells were treated with Cytochalasin D (CytD, 0.3 µM, 24 h) to inhibit actin polymerization. **k** Shown are representative fluorescence microscopy images of phalloidin stained cells. Images were acquired and are displayed using identical settings. Scale bar is 10 µm. **l** Quantification of the experiment representatively shown in (**k**) by fluorescence intensity measurements (**l**). *N* = 60–100, *n* = 4. Statistical analysis was done with a two-tailed paired *t* test. *p < 0.05. **m** Quantification of the experiment representatively shown in (**k**) by MSRE-qPCR. *n* = 3. Statistical testing was performed using a two-tailed unpaired *t* test. *p < 0.05. **b**, **c**, **f**, **g**, **I**, **j**, **l**, **m** In the dot blots, each dot is the mean value obtained for one biological repeat, the line indicates the mean of all biological repeats, and error bars represent their standard deviation. Paired measurements are indicated with the color coding.

Cell-cell contacts are among the most notable physiological features that are different between high- and low-density epithelial cultures. While MCF10A cells do not form tight junctions under standard cell culture conditions^23,24^, they form E-cadherin based adherens junctions. E-cadherin builds calcium-dependent homophilic interactions, which are important for the maintenance of epithelial tissue integrity^25^. To test the involvement of E-cadherin in the observed DNA methylation changes, we performed calcium depletion experiments. To this end, MCF10A-BiAD cells were seeded at high density in calcium-free growth medium and the DNA methylation level of α-satellite repeats was measured 24 h later. As expected, calcium depletion impaired the formation of E-cadherin mediated adherens junctions (Fig. 3a). Notably, this led to an increase of α-satellite methylation by comparison to cells cultured under matching densities but in normal medium (Fig. 3a–c). Methylation differences with and without calcium were not observed in low density cultures (Fig. S4a–c). Calcium is a broad cellular signal^26,27^ and the effects on DNA methylation could stem directly from E-cadherin perturbation or could be indirect, downstream of calcium ion channels. To target E-cadherin (CDH1) directly, a siRNA-mediated knock-down of this gene was performed and confirmed by Western blot (Fig. 3d) and immunofluorescence staining (Fig. 3e). Importantly, upon reseeding of cells two days post transfection, DNA methylation at α-satellite repeats was higher in E-cadherin-depleted cells compared to the levels seen in the control siRNA cells. This effect was observed by both microscopy (Fig. 3e, f) and MSRE-qPCR (Fig. 3g) and was specific to cells at high cell density (Fig. S4d–f).

We next employed an E-cadherin blocking antibody to acutely perturb formation of adherens junctions upon seeding of the cells^28^ (Fig. 3h, right panel). In line with the other perturbation experiments, antibody-mediated E-cadherin internalization resulted in a DNA methylation increase in high (Fig. 3h–j), but not in low density cultures (Fig. S4g, h). E-cadherin mediates signal transmission from the cell membrane to the nucleus in association with the actin cytoskeleton^29^. We therefore inhibited actin polymerization by treating the confluent cells with Cytochalasin D (CytD) for 24 h before imaging (Fig. 3k). Actin disruption as evidenced by phalloidin staining (Fig. 3k, lower panel) resulted in an increase in the DNA methylation of repeats, which was seen by both microscopy and methylation sensitive restriction digestion (Fig. 3l, m).

To investigate if the link between E-cadherin and DNA methylation is conserved in other cell lines, we performed experiments in MCF7 cells, a breast cancer cell line known to form strong E-cadherin-positive adherens junctions^30,31^ (Fig. S5a). MSRE-qPCR was employed to directly interrogate the levels of DNA methylation at α-satellites in this cell system. Also here, α-satellites displayed high DNA methylation turnover as treatment with 2 µM 5-aza-dC resulted in around 50% reduction of DNA methylation already after 24 h (Fig. S5b). In line with our findings in MCF10A cells, high density MCF7 cultures had lower DNA methylation levels by comparison to low density conditions (Fig. S5c). The remodeling of repeat DNA methylation was dependent on intact adherens junctions as demonstrated by E-cadherin inactivation through either siRNA knock-down (Fig. S5d–f) or the E-cadherin blocking antibody (Fig. S5g). Together, these experiments indicate that the communication between adherens junctions and repeat DNA methylation extends beyond the MCF10A system.

### Cell density dependent 5mC changes regulate α-satellite transcription

**Fig. 4:**
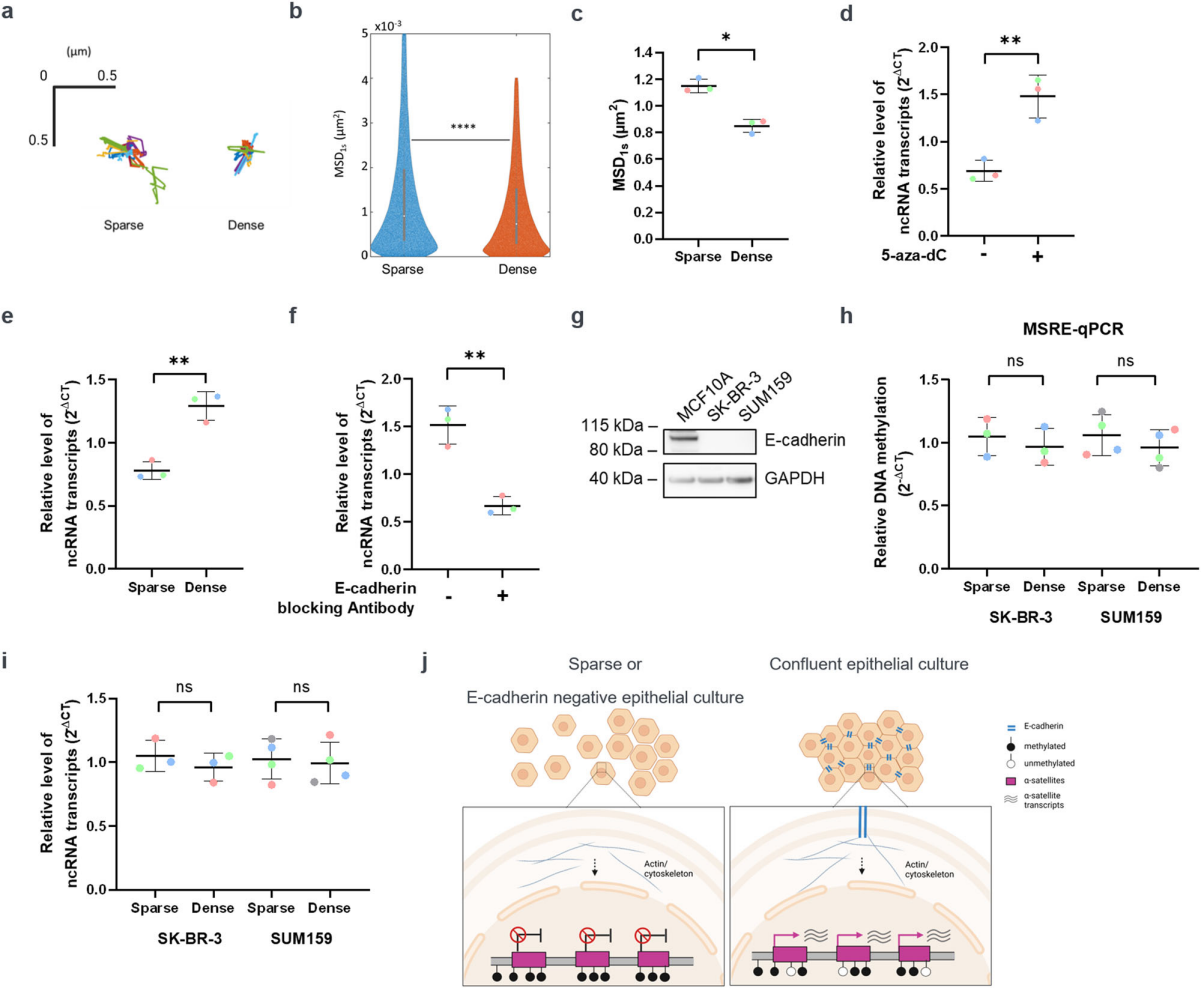
Cell density-dependent 5mC changes alter local chromatin mobility and α-satellite transcript expression. **a**–**c** Mean square displacement (MSD) of mVenus spots was measured in sparse and dense MCF10A-BiAD cultures. **a** Shown are representative trajectories integrated over a lag time of 3 s. *N* = 20. **b** Representative violin plot showing the distribution of mVenus spot movement (lag time of 1 s) in data collected from one biological repeat for sparse (blue) and dense (orange) seeded cells. *N* = 50–140, statistical testing was determined with a two-tailed unpaired *t* test, ****p < 0.0001. **c** Quantification of the MSD experiments representatively shown in (**b**). *n* = 3, *N* = 50–140. Statistical analysis was done with a two-tailed paired *t* test, *p < 0.05. **d** RT-qPCR analysis of α-satellite transcripts showing increased transcription in 5-aza-dC 24 h after drug treatment. *n* = 3. Statistical analysis was done with a two-tailed unpaired *t* test, **p < 0.01. **e** RT-qPCR analysis of α-satellite transcripts was performed on RNA isolated from MCF10A cells grown for 24 h in either sparse or confluent conditions. n = 3. Statistical analysis was done with a two-tailed unpaired *t* test, **p < 0.01. **f** RT-qPCR was used to analyze transcription from α-satellite DNA in MCF10A cells that had been treated for 24 h with the E-cadherin blocking antibody. *n* = 3. Statistical analysis was done with a two-tailed unpaired *t* test, **p < 0.01. **g** Western blot analysis of cell lysates isolated from MCF10A, SK-BR-3 and SUM159 cells and probed for E-cadherin. GAPDH was used as loading control. **h** MSRE-qPCR analysis of the α-satellite locus on genomic DNA isolated from SK-BR-3 and SUM159 cells, which were cultured for 24 h under either sparse or dense conditions. *n* = 3. Statistical analysis was done with a two-tailed unpaired *t* test, ns not significant. **i** qPCR analysis of the α-satellite transcripts on RNA isolated from SK-BR-3 and SUM159 cells, which were cultured for 24 h under either sparse or dense conditions. *n* = 3. Statistical analysis was done with a two-tailed unpaired *t* test, ns: not significant. **j** Scheme of newly discovered pathway linking the methylation status and transcription state of satellite DNA to cell density, mediated by E-cadherin. The figure was generated using Biorender. **c**, **d**, **e**, **f**, **h**, **i** In the dot blots, each dot is the mean value obtained for one biological repeat, the line indicates the mean of all biological repeats, and error bars represent their standard deviation. Paired measurements are indicated with the color coding.

In response to external mechanical forces like confluency^32,33^, compression, shear stress or stretching, cells adapt by engaging mechanoresponsive signaling pathways^26,34–36^, which can ultimately modulate chromatin and nuclear mechanics^26,37^. We therefore investigated if the DNA methylation changes that we observe in response to cell culture density impact chromatin mechanics. For this, we utilized the live cell imaging ability of the BiAD sensor and probed the rheology of chromatin specifically at the methylated repeats. To this end, we recorded the mVenus signal with a rate of 100 ms/frame and quantified its mean square displacement (MSD) over time (Fig. 4a). Analysis integrating the MSD over a short lag time of one second revealed a slower movement of the methylated repeats, indicative of a more restrictive local chromatin environment when the MCF10A-BiAD cells were cultured under dense conditions (Fig. 4b, c). Active transcription was reported to locally constrain chromatin movement^38^ and α-satellite DNA can give rise to ncRNAs under specific cell state conditions^39,40^. The expression of these transcripts is controlled by chromatin modifications^41^. Indeed, 5-aza-dC treatment for 24 h lead to an increase in α-satellite transcription in MCF10A cells (Fig. 4d). Notably, more ncRNA transcripts were observed in dense cultures (Fig. 4e). Reciprocally, E-cadherin antibody treatment of confluent cells decreased the levels of these transcripts, confirming the involvement of adherens junctions in the regulation of α-satellite transcription (Fig. 4f).

The signaling pathway discovered here is dependent on E-cadherin expression. As E-cadherin is often altered or absent in cancer^42^, the two breast cancer cell lines SK-BR-3 and SUM159, which lack E-cadherin (Fig. 4g), and are not contact inhibited^43,44^ were examined. In both, sparse and dense conditions, DNA methylation and α-satellite transcription remained unchanged, highlighting the necessity of E-cadherin for adjustment of DNA methylation upon cell density (Fig. 4h, i).

### Cell density dependent 5mC turnover does not rely on the activity of TET enzymes

We were next interested to identify the enzymes responsible for the 5mC turnover under our experimental conditions. While DNMTs catalyze the transfer of the methyl group to DNA, TET demethylases are responsible for the active removal of this mark^3^. We first performed Western blot experiments to test if cell culturing conditions impinge on the total expression levels of these enzymes. No change in DNMT1, DNMT3A or TET1 protein levels could be observed among the sparse and densely seeded cells (Fig. S6). This suggests that changes in enzymatic activity and not in protein abundance regulate the 5mC turnover. The fact that α-satellite repeats are less methylated in dense cultures could reflect a higher rate of local DNA demethylation. Indeed, in mouse cells, TET1 was reported to be essential for the 5mC to 5hmC conversion at pericentromeric heterochromatin^45^. We therefore used Bobcat339^46^, an established inhibitor of TET1/2, to investigate if inhibiting the activity of these enzymes in high density cultures increases the signal of the BiAD sensor. Of note, Bobcat339 activity was recently suggested to be enhanced by the amount of Cu(II) in the drug preparation^47^. A 24 h treatment of MCF10A cells with 10 µM Bobcat339 resulted in an approximately 50% increase in global DNA methylation levels, as based on 5mC staining (Fig. S7a, b). However, no change in the mVenus signal could be observed under these conditions (Fig. S7c, d). This suggests that TET enzymes are not involved in the regulation of α-satellite 5mC turnover in response to culture density. Instead, the transmission of information from adherens junctions to repetitive DNA methylation might rely on the local fine-tuning of DNMT activity, as suggested by the response to 5-aza-dC.

## Discussion

Repetitive elements like α-satellite arrays are considered archetypical sites of silenced heterochromatin. Nevertheless, the fact that these sequences can be actively transcribed^7,9^ and their methylation levels change within hours after treatment with DNMT inhibitors^12^, suggest that the DNA methylation markup of repeats is not static but is subject to local active turnover. Due to technical challenges associated with their repetitive nature, the link between the DNA methylation of repeats and the phenotypic state of cells, has remained largely unexplored^2^. By using a fluorescent CRISPR/dCas9 reporter of DNA methylation^16^ we here uncovered a signaling pathway connecting epithelial adherens junctions to the DNA methylation levels and transcriptional output of α-satellite repeats (Fig. 4j). These microscopy-based findings were confirmed by flow cytometry as well as direct profiling of DNA methylation at α-satellites by MSRE-qPCR. Importantly, while the BiAD sensor recognizes α-satellite arrays primarily enriched on chromosome 9, our sequence analysis revealed clustered sgRNA binding sites also on other chromosomes (Supplementary Data 1). Moreover, the methylation-sensitive digestions^16^ and the amplified ncRNA transcripts^41^ are a readout for α-satellite repeats present also on other human chromosomes. This allows us to generalize our observations to a broader context. At a molecular level, we found that junctional E-cadherin, which links to the actin cytoskeleton, is required for signal relay to the nucleus. Accordingly, siRNA-mediated E-cadherin depletion, E-cadherin internalization triggered by a blocking antibody or actin depolymerization by Cytochalasin D rescued the decrease of DNA methylation observed in cells cultured at high density. This phenomenon was observed in MCF10A and MCF7 breast epithelial cells, both of which form E-cadherin-positive adherens junctions, indicating a conserved mechanism. Of note, while our work is centered on adherens junctions, it is possible that the signaling pathway we identified also extends to other junctional structures such as tight junctions. This will be interesting to explore in follow up investigations.

To our knowledge, this work is the first to functionally link adherens junctions to the DNA methylation state of repetitive elements. Our results reinforce our growing understanding of the role of epithelial integrity in the regulation of epigenetic pathways and chromatin states. In line with our findings, certain mechanical perturbations have been reported to remodel the repressive chromatin marks at constitutive heterochromatin in epithelial cells. For example, in scratch-wound assays of mouse melanoma cells, a global increase in chromatin condensation was reported to support nuclear movement, cell migration and thus efficient monolayer closure^48^. In another study^26^, cell monolayers subjected to uniaxial stretch showed a transient loss of H3K9me3 modifications at non-coding heterochromatin. This response was not correlated with transcriptional changes but promoted nuclear softening and was proposed to protect the genome from mechanical stress. Upon prolonged stretching, the mechanical energy was redistributed to cell-cell contacts and the initial chromatin state was restored. In our study, at high cell density reduced repeat DNA methylation, decreased local chromatin mobility at α-satellites and favored their transcription. Strikingly, this coupling between cell density and α-satellite transcription was detectable only in contact inhibited cell lines and not for the SK-BR-3 and SUM159 cells. Since α-satellite transcription is important for cell division coordination^10^, we hypothesize that this E-cadherin driven pathway could control proliferation rates as epithelial cultures approach confluence. Because the size of the nucleus was not affected under our experimental conditions, compression does not appear to be the driving force of chromatin remodeling at DNA repeats. Instead, this could be an early signaling response that proceeds the final stage of epithelial tissue maturation and which is characterized by cell cycle inhibition, reduced cell motility and high mechanical tension.

In addition to these mechanical implications, cell junction integrity was also linked to the chromatin state at specific gene promoters. Recently, Bhatt et al. ^49^ reported an increased DNA methylation at the caspase-8 promoter in keratinocytes during wound healing. The authors linked this to cellular tension, which was found to modulate the subcellular localization of the de novo methyltransferase DNMT3A. Accordingly, while DNMT3A was localized in the cytosol in confluent monolayers, disruption of cell-cell contacts by EGTA treatment resulted in DNMT3A shuttling to the nucleus. A reduction in the nuclear localization of DNMT3A with increasing cell density was also observed in smooth muscle cells^50^. Although our DNA methylation findings align with these results, our preliminary investigations did not reveal any alterations in the subcellular distribution of DNMT3A. This indicates that alternative mechanisms are in place to fine tune the local activity of DNMTs. For example, casein kinase 2 mediated phosphorylation of DNMT3A was reported to inhibit the activity of the enzyme and redistribute the protein from heterochromatic repeats to euchromatic genomic regions^51^. Furthermore, recently it was found^52^ that DNMT1 undergoes O-GlcNAcylation under hyperglycemic conditions. This modification results in a widespread reduction in DNA methylation, except at young transposable elements. Exploring the regulatory mechanisms of DNMTs in response extracellular cues is a promising avenue of research.

In conclusion, in this study we identified a signaling pathway linking epithelial cell junction integrity to the chromatin landscape. We anticipate that our findings will not only help better understand epithelial tissue homeostasis but also have implications for cancer development, which is marked by a loss of adherens junctions, lack of contact inhibition and high epigenetic instability^2,53^.

## Material and methods

### Cell culture

MCF10A cells (provided by Prof. Andreas Hecht, University of Freiburg) were cultured in DMEM/Ham’s F12 (Gibco) supplemented with 5% horse serum (Gibco), 20 ng/mL epidermal growth factor (EGF, R&D), 500 ng/mL hydrocortisone (Sigma Aldrich), 10 µg/mL insulin (Sigma Aldrich) and 100 ng/mL cholera toxin (Sigma Aldrich). Resuspension medium was DMEM/Ham’s F12 (Gibco) supplemented with 20% horse serum (Gibco). MCF7 cells (provided by Dr. Angelika Hausser, University of Stuttgart) were maintained in RPMI medium (Gibco) supplemented with 10% fetal calf serum (Gibco). SUM159 (provided by Dr. Thordur Oskarsson, DKFZ) and SK-BR-3 cells (CLS Cell Lines Service GmbH) were cultured in DMEM medium (Gibco) with 10% fetal calf serum (Gibco). LentiX HEK293 cells (provided by Dr. Philipp Rathert, University of Stuttgart) were maintained in DMEM medium (Gibco) with 10% fetal calf serum (Gibco), 10 mM HEPES (Thermo Fisher Scientific) and 1 mM Sodium Pyruvate (Thermo Fisher Scientific). The cells were grown at 37 °C in a saturated humidity atmosphere containing 5% CO_2_. All cell lines were authenticated by STR profiling, tested negative for Mycoplasma (Lonza) and were kept in culture for no longer than 2 months. Cells were seeded in sparse, 5 × 10^3^ cells/cm^2^, or dense conditions, 10^5^ cells/cm^2^. Analysis via immunofluorescence, flow cytometry, Western blotting or DNA methylation readout was done 24 h after seeding.

### Cloning and generation of cell lines with stable expression of BiAD modules

For the generation of the MCF10A cells with stable expression of the BiAD sensor, the BiAD 3 modules, which recognize methylated DNA of α-satellite repeats enriched on human chromosome 9 (Ch9), were subcloned from the transient transfection plasmids used by ref. ^16^ into lentiviral backbones. In brief, the sgRNA sequence (5′-TGGAATGGAATGGAATGGAA-3′)^16,20^ was subcloned into the lentiGuide-Puro vector (Addgene #52963) that had been digested with BsmBI (Thermo Scientific). The Flag-MBD-VenC detector module was amplified by PCR using the primers pLV_Flag_fw 5’-CCATTTCAGGTGTCGTGAGGATCATGGACTATAAGGACCACGACGG-3’ and pLV_VenC_rev 5’-CCGCCCTCGAGGAATTTTACTTGTACAGCTCGTCCATGCC-3’ and subcloned via Gibson assembly (NEB) into the pLV-EF1a-IRES-Blast vector (Addgene #85133) that had been digested with EcoRI and BamHI (NEB). The pHAGE-TO-dCas9-VenN and pHAGE-TO-dCas-Venus has been previously described in ref. ^16^. The identity of all constructs was confirmed by Sanger sequencing.

For the production of lentiviral particles, LentiX HEK293T cells were transfected with the individual vectors described above and the packaging plasmid psPAX2 (Addgene #12260) and pCMV_VSV-G (Addgene #8454). After determining the multiplicity of infection (MOI), the viruses were mixed at a ratio of 1:1:5 for MBD: dCas9: sgRNA and MCF10A cells were transduced corresponding to a MOI of 1. The cells were selected 48 h after infection using 1.5 µg/mL puromycin (Sigma Aldrich) and 10 µg/mL blasticidine (Gibco). Single cell clones were obtained via flow cytometry. The successful expression of the Flag-MBD–VenC and dCas9-VenN modules was assessed by immunofluorescence and Western blotting. To ensure expression of the sgRNA, the culture medium was supplemented with puromycin during cell line propagation. Of note, since imaging of the MCF10A-BiAD cells revealed secondary smaller mVenus spots in addition to the clusters expected for the two chromosome 9, the sgRNA sequence was blasted against the hg38 in the Cas-OFFinder^54^ database. This analysis revealed additional clustered and specific sgRNA binding sites on other chromosomes. This is in line with the fact that α-satellites are present also on other human chromosomes^7^. We have uploaded a list with the first 1000 hits of the sgRNA blast, sorted by chromosome number, as Supplementary Data 1.

### Inhibitor treatment

To reduce DNA methylation in MCF10A or MCF7 cells, 5-aza-dC (Jena Bioscience, cat. no. N-DN-1771-10) treatment was performed for 24 h at a final drug concentration of 0.25 µM for MCF10A cells or 2 µM for MCF7 cells, respectively. The drug was dissolved in 50% acetic acid at 10 µM and replaced on a daily basis. As a control, an equal volume of solvent, was added to the cell culture medium.

To inhibit actin polymerization, Cytochalasin D (CytD, Enzo, cat. no. BML-T109-001) was used for 24 h at a final drug concentration of 0.3 µM. The drug was dissolved in DMSO at 5 mM and was added to the cell culture medium 24 h after cell seeding. As a control, an equal volume of solvent was added to the cell culture medium.

Bobcat339 (TETi, Selleckchem, cat. no. S6682) treatment was used to inhibit TET1/TET2 enzymes. The drug was dissolved in DMSO and was added to the cell culture medium 24 h after cell seeding. An equal volume of solvent was used as control. The final concentration of Bobcat339 used for the treatment is annotated in the corresponding figure.

### Calcium depletion assay

MCF10A-BiAD cells were seeded sparse or confluent in an appropriate vessel. Medium was changed 24 h later to DMEM medium without calcium and magnesium (-CaCl_2_ - MgCl_2_, Gibco) for calcium depletion. To remove residual calcium, the cells were washed once with PBS without calcium and magnesium. As a control, DMEM medium containing CaCl_2_ and MgCl_2_ (Gibco) was used. Analysis via immunofluorescence or MSRE-qPCR was done 24 h after medium change.

### E-cadherin blocking experiments

MCF10A-BiAD or MCF7 cells were seeded sparse or dense in an appropriate vessel. While seeding, 5 µg/mL of the E-cadherin blocking antibody SHE78-7 (Zymed, cat. no. 13-5700, lot. no. 49567 A) was added directly to the cells. Analysis via immunofluorescence or MSRE-qPCR was done 24 h after the seeding.

### E-cadherin knock-down experiments

To silence E-cadherin in MCF10A-BiAD or MCF7 cells a small interfering RNA inhibiting E-cadherin (CDH1) (Thermo Fisher s2768) was used. Silencer select control siRNA (NT, ambion by life technologies, 43900843) was used as a negative control. Therefore, 500,000 cells were seeded in a 6-cm cell culture dish (Greiner Bio-One). A total of 5 nM siRNA was transfected into the cells using 10 µL Lipofectamine RNAiMax Reagent (Invitrogen) in 1 mL OptiMEM (Gibco). After 48 h the cells were seeded sparse or dense and analyzed via immunofluorescence, Western blotting or MSRE-qPCR 24 h later.

### Cell lysis, SDS-PAGE and Western blotting

Cells were lysed in RIPA buffer (50 mM Tris-HCl pH 7.5, 0.1% [w/v] SDS, 1% NP40, 0.25% [v/v] sodium deoxycholate, 150 mM NaCl, 0.5 mM PMSF, 1 mM sodium orthovanadate, 10 mM sodium fluoride, 20 mM β-glycerophosphate and cOmplete™, EDTA-free Protease Inhibitor Cocktail [Roche]) for 15 min on ice. The lysate was then centrifuged at 16.100 xg, 4 °C, 15 min and the resulting supernatant was quantified using the DC Protein Assay (Bio-Rad). For protein expression analysis, the clear lysate was separated on a 4–12% Bis-Tris Gel (NuPAGE, Thermo Fisher) followed by transfer onto a nitrocellulose membrane using an iBlot® device (iBlot®Gel Transfer Stacks; Invitrogen). The membranes were blocked with 0.5% (v/v) blocking reagent (Roche) in PBS containing 0.05% (v/v) Tween-20 and 0.01% (v/v) Thimerosal and then incubated with primary antibodies, overnight at 4 °C, followed by 1 h incubation with HRP-conjugated secondary antibodies at room temperature. The chemiluminescent signal was detected using an AmershamTM Imager 600 device (GE Healthcare) followed by quantification of the 16-bit images in the linear range using the inbuilt ImageQuant TL 8.1 software.

### Antibodies

The following primary antibodies were used for detection: anti- β-Catenin (BD, # 610154, 1.0 mg/mL, IF 1:500), 5-methylcytosine (5-mC) (D3S2Z) (Cell Signaling, #28692 S, IF 1:1600), anti-Cas9 (Santa Cruz, sc-517386, 0.2 mg/mL, IF 1:100, WB 1:250), anti-DNMT3A (64B1446) (Novus Biologicals, NB120-13888SS, 1.0 mg/mL, WB 1:1000), anti-DNMT1 (abcam, ab188453, 1.1 mg/mL, WB 1:1000), anti-E-cadherin (Cell Signaling, #3195, 0.2 mg/mL, WB 1:1000, IF 1:200), anti-Flag M2 (Sigma Aldrich, F1804, 1.0 mg/mL, WB 1:1000), anti-DYKDDDDK Flag tag (D6W5B) (Cell Signaling, #14793, IF 1:100), anti-GAPDH (Sigma Aldrich, G9545, 1.0 mg/mL, WB 1:5000), anti- α-Tubulin (Millipore, #05-829, 1.0 mg/mL, WB 1:10000), anti-TET1 (E5F1O) (Cell Signaling, #40142, WB 1:1000).

For Western blots, HRP-labeled secondary anti-mouse-IgG and anti-rabbit-IgG were obtained from Dianova (#115-035-062, #111-035-144, 1:10.000). For immunofluorescence, Alexa-Fluor®-labeled secondary IgG antibodies from Thermo Fisher Scientific (#A32727, #A32733, 1:1000) were used. Alexa-Fluor®-labeled Phalloidin (Molecular Probes, A222284), SPY650-DNA (Spirochrome, SC501) and DAPI (Sigma Aldrich, D8417) were used for fluorescence microscopy.

### Immunofluorescence

To investigate the signal intensity of the complementation signal, MCF10A-BiAD cells were seeded on Collage R-coated (Serva, cat. No. 47254.01) microscopy cover slips. 24 h after seeding or treatment, the slides were washed with MgCl_2_ and CaCl_2_ containing PBS (Gibco), followed by crosslinking with 4% formaldehyde solution (Gibco) for 15 min at room temperature. For immunofluorescence staining, the cells were permeabilized with 0.2% Triton X-100 (Roth) for 5 min at RT and blocked with 2% BSA solution pH 7.5 (Roth, cat. No. 8076.2, Lot 169283077) for 1 h at room temperature. This was followed by incubation with primary antibodies overnight, at 4 °C and then secondary antibody incubation for 1 h at room temperature. For the E-cadherin blocking antibody experiments, after fixation and blocking with BSA, the cells were incubated with a 1:200 dilution of the anti-E-cadherin rabbit monoclonal antibody (Cell Signaling, #3195) in 2% BSA overnight, at 4 °C. This was followed by secondary antibody staining as described above. For the 5-methylcytosine staining, after fixation, the cells were subjected to a 30 min denaturation step with 2 N HCl. This was followed by 10 min neutralization with 100 mM Tris/HCl, pH 8.5. The blocking step was extended to 2 h.

Slides were mounted in ProLong® Gold Antifade Reagent (Cell Signaling) and left for curing overnight at room temperature. The slides were imaged on an LSM 980 Airyscan Zeiss confocal microscope equipped with a Plan-Apochromat 63 × /1.40 Oil DIC M27 objective and using the ZEN 3.2 software. The thickness of image slices was 1.2 µm with 7 slices (Z step of 200 nm). The laser excitation wavelengths as well as emission collection windows were used as follows: 405 nm and 300–720 nm for DAPI; 514 nm and 495–548 nm for mVenus; 561 nm and 380–608 nm for Alexa-Fluor®-555 coupled probes; 639 nm and 659–720 nm for Alexa-Fluor®-647 coupled probes.

### Image analysis

Post-acquisition, the images were processed for visualization using the ZEN light (cropping and maximum intensity projections) or the Fiji software (pseudo coloring and image annotations). Image analysis was performed using a semi-automated combined CellProfiler^55^ and Python (version 3.12.0) script generated in house. In brief, the analysis pipeline focused on identifying cell nuclei based on the DAPI staining. Within the identified nuclei, the intensity of the mVenus channel was used to create a second mask for the Ch9 spots, from which the mean fluorescence intensity was extracted. To assess the background fluorescence of each cell nucleus, the Ch9 spot mask was subtracted from the nucleus mask, and the remaining area was used to calculate the mean background mVenus signal intensity. This value was then subtracted from the mVenus values obtained for Ch9 spots on a cell-by-cell basis.

To measure the nuclear area, the MCF10A cells were incubated with SPY650-DNA (Spirochrome) according to the instructions of the manufacturer. Images were acquired on an LSM 980 Airyscan Zeiss confocal microscope equipped with a Plan-Apochromat 63 × /1.40 Oil DIC M27 objective and were processed using an automated CellProfiler pipeline. To this end, the maximum fluorescence intensity of 7 consecutive confocal slices (total thickness of 1.2 μm) was used to generate orthogonal projections. The resulting images were smoothed by a Gaussian filter (artifact diameter of 10 pixels), followed by the definition of the area occupied by the SPY650-DNA signal per image. This value was divided by the number of nuclei in the image to obtain the mean value of the nuclear area. Nuclei in contact with the image borders were excluded. This analysis was performed with 10 images per experiment and condition.

### Live-cell imaging and mean square displacement analysis

Cells were seeded in a Collage R-coated (Serva, cat. No. 47254.01) 4-compartment glass bottom dish (Greiner Bio-One). Imaging was started 24 h later. Time-lapse videos (frame rate 0.1 s/frame) were recorded on a spinning Disk Microscope (Zeiss AxioObserver SD) equipped with Yokogawa CSU-X1 Spinning Disk Unit and using the ZEN vs 2.3 software. The raw videos were first processed by a moving average over every 5 adjacent frames. A MATLAB code^56^ was then used to track the mVenus spots in the videos, and two-dimensional Cartesian coordinates ${{{{{\boldsymbol{r}}}}}}(t)$ as a function of time $t$ of each spot were recorded. Further, the MSD (mean squared displacement) as a function of lag time $\tau$ was calculated as

$${MSD}\left(\tau \right)=\left\langle {\left|{{{{{\boldsymbol{r}}}}}}\left(t\right)-{{{{{\boldsymbol{r}}}}}}\left(t+\tau \right)\right|}^{2}\right\rangle ,$$

where $< >$ is an average over both time $t$ and all the tracked trajectories. Outliers, defined as values with more than three scaled median absolute deviations from the median were removed before data plotting and statistical analysis.

### Flow cytometry

Cells were trypsinized and collected by centrifugation. The cell pellet was resuspended in PBS containing 10% Horse Serum. Data was collected by the BD FACSDiva software (Version 8.0.1) using the FACS Aria III system (BD Bioscience). Post-acquisition analysis was performed with the FlowJo software (v10.7.2). Median fluorescence intensity of the mVenus signal was measured. For single cell sorting, the cells were sorted into a 96-well plate based on the fluorescence intensity of the complementation signal measured via the FITC channel.

For cell cycle analysis, the cells were subjected to FxCycle^TM^ Violet staining (Invitrogen), following the instructions of the manufacturer. Briefly, the cells were trypsinized as above, washed with ice cold PBS and the cell pellet was resuspended in 70% ice-cold ethanol by vigorous vortexing. Following 30 min fixation of ice, the cells were spun down, washed twice with PBS and resuspended in the FxCycle^TM^ Violet staining solution. After 30 min incubation at room temperature, the FxCycle^TM^ Violet signal was measured on a MACSQuant® VYB (Miltenyi Biotec) device using a 405 nm laser line and a 450/50 nm filter. Post-acquisition analysis was performed with the FlowJo software (v10.7.2).

### Methylation sensitive restriction enzyme digestion coupled with RT-qPCR (MSRE-qPCR)

24 h after cell seeding, total genomic DNA was isolated using the PureLink Genomic DNA Mini Kit (Invitrogen). 600 ng of the resulting material was fragmented with XbaI (NEB) to increase target site accessibility. The 5mC-inhibited enzyme AciI (NEB) was used for the recognition of methylated CpG sites. After digestion, the DNA was cleaned up using the NucleoSpin Gel and PCR Clean-up Kit (Marcherey-Nagel). Quantitative PCR was performed in a CFX96 Real-Time System (Bio Rad) using the *Power* SYBR® Green RNA-to-CT™ 1-Step Kit (Thermo Scientific). The MSRE-qPCR strategy is shown in Figure S2. In brief, two sets of primers were designed based on the GRCh38 57999639 - 58000359 α-satellite locus previously used by ref. ^16^ to validate the BiAD method. Asat_MSRE_fw 5’-CGGACTGCAGTGGCTCAATC-3’ and Asat_MSRE_rev 5’-AAAGAGAGGTTCAGCTGGGC-3’ flank six AciI recognition sites. The Asat_Ctrl_fw 5’-GGATATTTGGACCACTTTGAGGC-3’ and Asat_Ctrl_rev 5’-TGGGCGACAGAGCGAGAC-3’ are located within the repeat sequence but do not contain AciI motifs and were used for input normalization. The reaction mixture (10 µL) contained 5 µL SYBR Green, 300 nM Primer and 5 ng of the digested DNA. Quantitative PCR was performed under the following conditions: 95 °C for 10 min, then 45 cycles with 95 °C for 15 s and 62 °C for 1 min. Following the final cycle, the melt curves of the PCR products were determined to verify the integrity of the PCR products. For the comparison of the real time assays the C_T_ values were determined using the standard settings of the Bio-Rad CFX Manager software package (version 3.1; Bio-Rad).

### RNA isolation

RNA was isolated using the NucleoSpin RNA Kit (Macherey Nagel). To remove residual DNA that could interfere with the readout of ncRNAs from α-satellite repeats, the RNA extraction was followed by an additional rDNase digestion in solution according to the manufacturers’ protocol. Therefore 1/10 volume of rDNase mixed with rDNase buffer was added to the RNA eluate and incubated for 10 min at 37 °C. This was followed by an ethanol precipitation step. 0.1 volume of 3 M sodium acetate, pH 5.2 (Roth) and 2.5 volume 100% ice-cold ethanol (Roth) was added to one volume of sample. Sample was mixed thoroughly and incubated for 1 h at −20 °C, followed by centrifugation 16.100 × g, 4 °C, 10 min. The RNA pellet was washed with 70% ethanol and after drying, the pellet was resuspended in RNase-free water (Macherey-Nagel).

### Quantitative Real-Time PCR (qRT-PCR) Analysis

The quantitative PCR was performed in a CFX96 Real-Time System (Bio Rad) using the *Power* SYBR® Green RNA-to-CT™ 1-Step Kit (Thermo Scientific) in a 96-well format. Primers for the analysis of α-satellite expression were Asat_fw 5’-CACTCTTTTTGTAGAATCTGC-3’ and Asat_rev 5’-AATGCACACATCACAAAGAAG-3’^41^, which were constructed according to the α-satellite consensus sequence^57^. RPLP0 was used as control for normalization with following primers: RPLP0_FP 5’-CTCTGCATTCTCGCTTCCTGGAG-3’ and RPLP0_RP 5‘-CAGATGGATCAGCCAAGAAGG-3’. The reaction mixture (10 µL) contained 5 µL SYBR, 300 nM Primer, 1x RT Primer Mix, and 50 ng RNA. Non-template controls for each primer pair were included as well as a SYBR Mix without RT enzyme. Quantitative PCR was performed under the following conditions: 48 °C for 30 min, 95 °C for 10 min, then 40 cycles with 95 °C for 15 s and 60 °C for 30 s. Following the final cycle, the melt curves of the PCR products were determined to verify the integrity of the PCR products. For the comparison of the real time assays the C_T_ values were determined using the standard settings of the Bio-Rad CFX Manager software package (version 3.1; Bio-Rad).

### Statistics and reproducibility

Data are presented as mean ± SD, each experiment was performed at least three times on independent days, unless specified otherwise in the figure legend. For the dot plots, each dot represents the average value obtained for one biological repeat, where paired measurements are shown in the same color. For each biological repeat, the raw average values of the two conditions to be compared, were normalized to the average value obtained for both conditions. ‘N’ refers to the number of cells and ‘n’ to the number of independent biological experiments. Significance between two groups was determined by a two-tailed *t* test. One-way ANOVA was used to compare three groups. Data were analyzed using GraphPad Prism 8.0.2. *p* Values: n.s., not significant: *p* > 0.05, *p < 0.05, **p < 0.01, ***p < 0.001, ****p < 0.0001. The exact *p* values are collected in Table S1. All attempts at replication of data contained in this manuscript were successful. The repeat (n) times are labeled in the figure legend and in Table S1. No sample size calculation was performed. Sample size was determined from similar experiments in the literature. For the MSD experiments, outliers, defined as values with more than three scaled median absolute deviations from the median were removed before data plotting and statistical analysis. No data were excluded from analysis for the other experiments. The work does not involve participant groups; therefore, randomization was not relevant to the study. The work does not involve participant groups; therefore, blinding was not relevant to the study.

### Reporting summary

Further information on research design is available in the Nature Portfolio Reporting Summary linked to this article.

### Supplementary information

Supplementary Information

Description of Additional Supplementary Files

Supplementary Data 1

Supplementary Data 2

Reporting Summary

## Data Availability

All numerical source data for the graphs shown in this article can be found in Supplementary data 2. Materials and all other primary data files are available upon reasonable request from the corresponding authors.
