# Supplementary material for: Repeat DNA methylation is modulated by adherens junction signaling
Source: Commun Biol. 2024 Mar 7;7:286. doi: 10.1038/s42003-024-05990-4 (PMC10920906; doi:10.1038/s42003-024-05990-4)
Supplement: Supplementary file 1 — Supplementary Information [file 42003_2024_5990_MOESM1_ESM.pdf]

Repeat DNA methylation is modulated by adherens junction signaling

Lisa-Marie Brenner[1], Florian Meyer[1], Haiqian Yang[2], Anja R. Köhler[3], Pavel Bashtrykov[3], Ming Guo[2], Albert Jeltsch[3], Cristiana Lungu[1,4,#], & Monilola A. Olayioye[1,4,#]

[1] Institute of Cell Biology and Immunology, University of Stuttgart, Allmandring 31, 70569 Stuttgart, Germany

[2] Department of Mechanical Engineering, Massachusetts Institute of Technology, Cambridge, MA 02139, USA.

[3] Institute of Biochemistry and Technical Biochemistry, University of Stuttgart, Allmandring 31, 70569 Stuttgart, Germany

[4] Stuttgart Research Center Systems Biology (SRCSB), University of Stuttgart, Nobelstraße 15, Stuttgart, Germany

[#]Correspondence: cristiana.lungu@izi.uni-stuttgart.de; monilola.olayioye@izi.uni-stuttgart.de

Supplementary Figures

Supplementary Figure 1: Characterization of MCF10A cells stably expressing the BiAD modules

Supplementary Figure 2: Scheme of the MSRE-qPCR strategy

Supplementary Figure 3: Flow cytometry analysis shows no difference in the cell cycle stage distribution among sparse and dense MCF10A cultures

Supplementary Figure 4: Functional inactivation of E-cadherin does not affect α-satellite DNA methylation in low density cells

Supplementary Figure 5: The methylation status of α-satellite sequences in MCF7 cells is sensitive to cell density

Supplementary Figure 6: Western blot analysis of DNMT and TET1 enzymes in cell lysates obtained from sparse and confluent MCF10A cultures

Supplemental Figure 7: TET inhibition does not alter the methylation status of repeats in confluent BiAD cells

Supplementary Figure 8: Uncropped Western blots

Supplementary Tables

Table S1: Summary of p values and sample sizes

Supplementary Data 1: List with the first 1000 hits of the sgRNA blast, sorted by chromosome number.

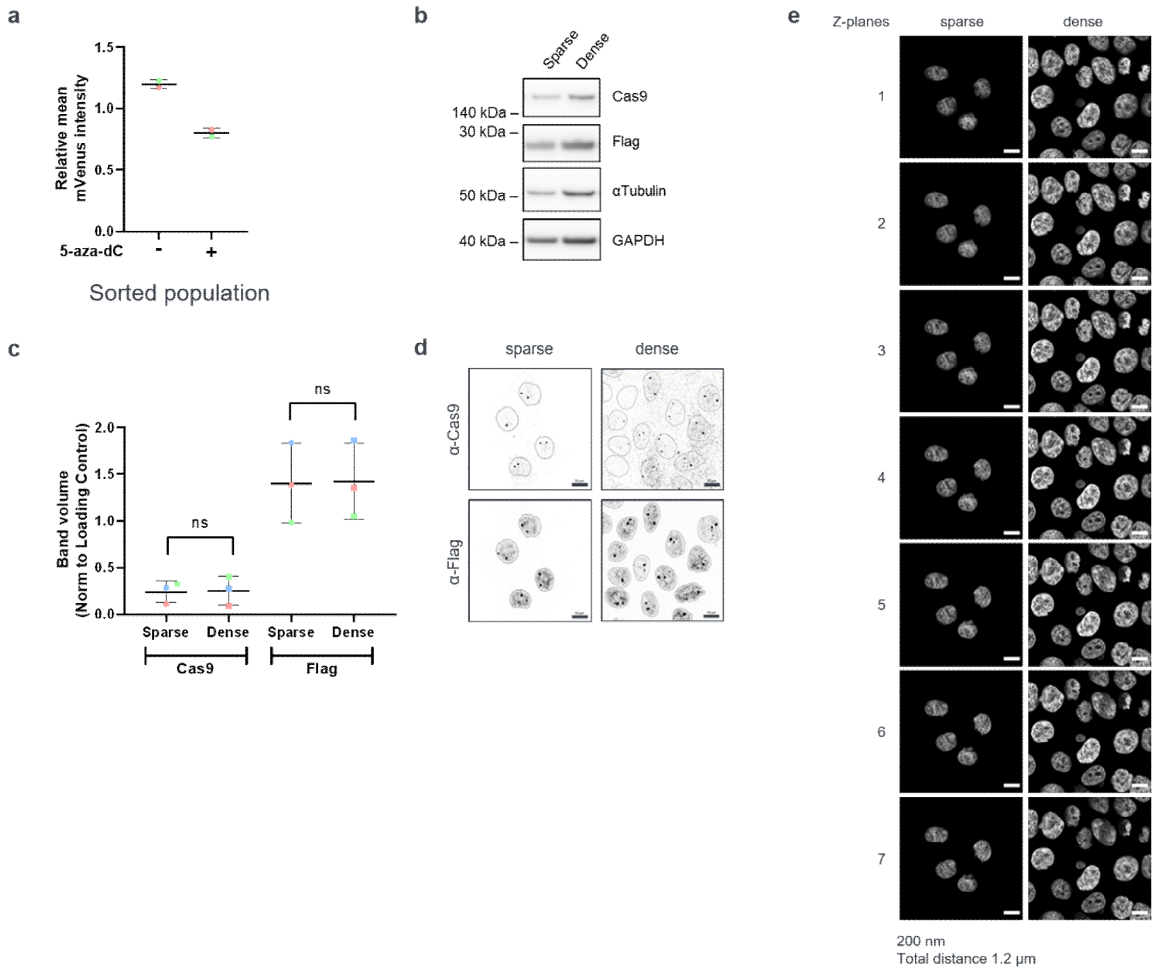

Figure S1: Characterization of MCF10A cells stably expressing the BiAD modules**. a:** A sorted pool of MCF10A cells expressing the BiAD modules was treated for 24 h with 5-aza-dC. Shown are quantification of immunofluorescent experiments (n= 2). N= 10 – 20. **b:** Western blot on lysate of the sorted MCF10A-BiAD reporter single cell clone confirming the expression of the BiAD modules in cells seeded under both sparse and dense conditions. α-tubulin and GAPDH were used as loading controls. Antibody probing for Cas9, Flag and αTubulin (re-probing of the anti-Flag blot) were performed on the same membrane strip, which was cut accordingly to the expected molecular weight of the target proteins. See also Figure S8D for the uncropped Western blot membranes. **c**: Densitometric quantification of the dCas9 anchor and the MBD detector protein expression levels as based on the Western blots representatively shown in (b). n= 3. Statistical analysis was done with a two-tailed paired t-test. ns: not significant. **d:** Representative fluorescence microscopy images of MCF10A-BiAD cells seeded under sparse and dense conditions. Primary antibodies against Cas9 and Flag confirmed the expression and localization of the dCas9 and Flag-MBD BiAD modules. DAPI was used to counterstain the nuclei and for nuclear mask definition (line annotation). Images were acquired and are displayed using identical settings. Scale bar is 10 µm. **e:** Representative fluorescence microscopy images of SPY-DNA-stained cells cultured under sparse and dense conditions. Shown are serial z-positions across the nuclei. Images were acquired and are displayed using identical settings. Scale bar is 10 µm. Distance between z-planes 200 nm, total distance 1.2 µm. (a, c) In the dot blots, each dot is the mean value obtained for one biological repeat, the line indicates the mean of all biological repeats, and error bars represent their standard deviation. Paired measurements are indicated with the color coding.

a

5'-GCTATTGGATATTTGGAGCACTTTGAGGCCTATGGTGGAAAAGGAAATATCTTCACAAAAAAAAACTAGAC
AGAAGCATTCTCAGAAACATCGTTCTGATGTGTGAATTCATCTCATAGAGTTGAACCTCTCTTTTTTTTTTTTT
TTTTGAGATGGAGTCTCGCTCTGTCGCCCAGGTCGGACTGCGGACTGCAGTGGCTCAATCTCAGCTCACTGC
AAGCTC**CG**CTTCCCGGGTTCATGCCATTCTCCTGCCTCAGCCTCCCGAGTAGCTGGGACTACAGGTGCC**CG**C
CAC**CG**CGCCCAGCTAATTTTATGTATTTTTAGTAGAGACGGGGTTTCACCTTGTTAGCCAGGATGGTCTCGAT
CTCCTGACCTCGTGATC**CG**CC**CG**CCTCGGCCTCCCAAAGTGCTGGGATTACAGGCGTGAGCCAC**CG**CGCCCA
GCTGAACCTCTCTTTTGAAAGA-3'

b

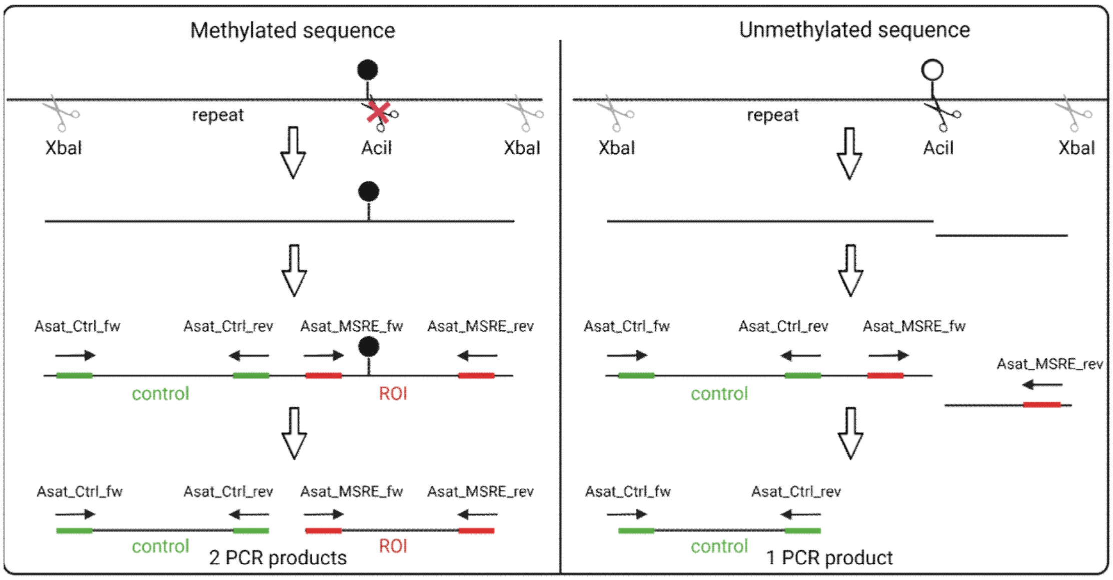

Figure S2: Scheme of the MSRE-qPCR strategy. a: Sequence of the α-satellite genomic locus based on the GRCh38 57999639-58000359 region. The AciI recognition motifs have been underlined and the corresponding CpG sites are shown in bold. Binding sites of the primers used for qPCR are shown in green (sequence does not contain AciI motifs) and in red (sequence contains 6 AciI motifs), respectively. **b**: Genomic DNA is fragmented with the restriction enzyme XbaI and with the methylation sensitive restriction enzyme AciI. (Left) Methylated CG sites (filled circles) within the AciI motif, block enzyme cleavage. This results in a DNA fragment that can be detected by PCR with the primers shown in red. (Right) If the AciI sites are not methylated (empty circle), the enzyme can cleave the substrate and no PCR product is obtained. The amplicon shown in green does not contain AciI motifs and is used as a control for data normalization. ROI: region of interest. This figure was generated using Biorender.

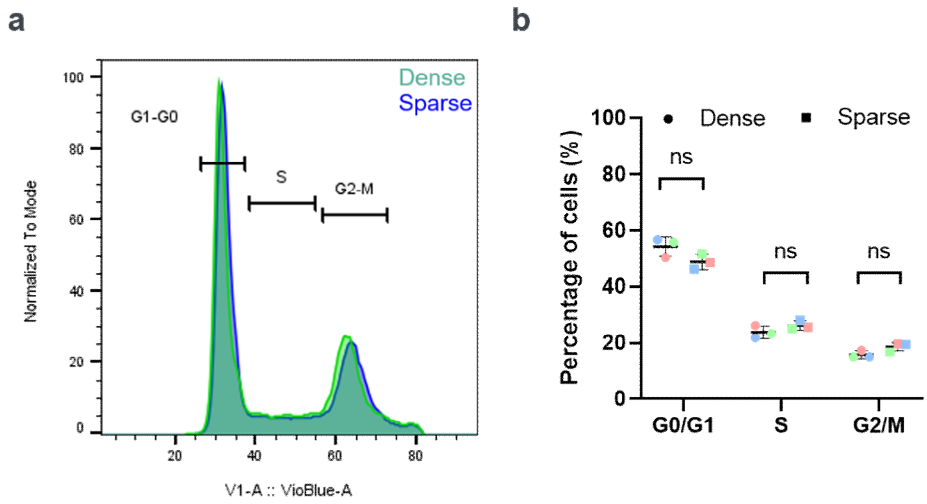

Figure S3: Flow cytometry analysis shows no difference in the cell cycle stage distribution among sparse and dense MCF10A cultures. a: MCF10A cells were grown for 24 h at either 5x 10^3 cells / cm^2 (sparse, blue) or 10^5 cells / cm^2 (dense, green) conditions followed by FxCycle™ Violet staining and cell cycle analysis by flow cytometry. The gating used to quantify the cell cycle stages is annotated on the histogram. **b:** Quantification of the flow cytometry experiments representatively shown in a. n=3. Statistical significance was determined by a two-way ANOVA followed by Sidak post-hoc testing.

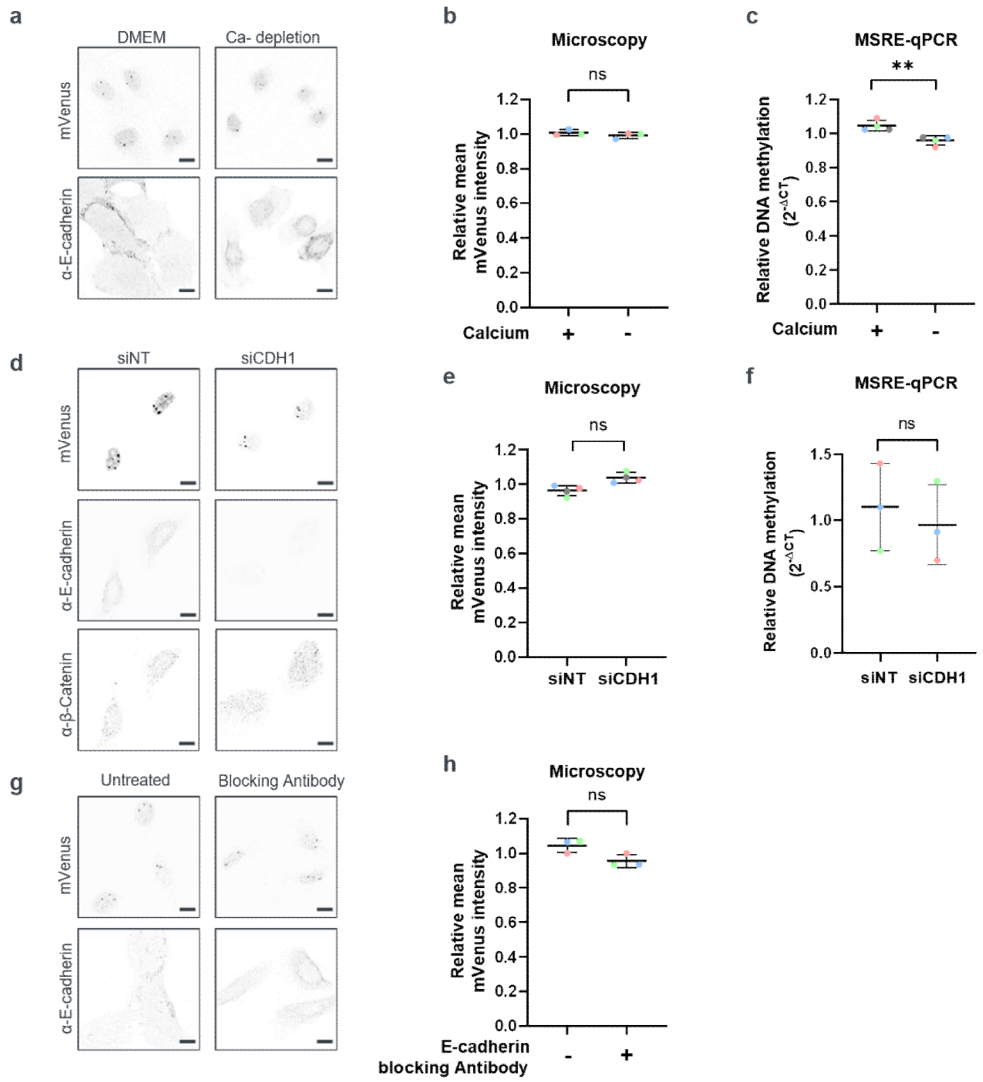

Figure S4: **Functional inactivation of E-cadherin does not affect α-satellite DNA methylation in low density cells. a:** Representative fluorescence microscopy images of sparsely seeded MCF10A-BiAD reporter cells grown for 24 h in either complete (left, DMEM) or low calcium medium (right, Ca^{2+} depletion). E-cadherin immunofluorescence staining was used to validate the absence of adherens junctions. Images were acquired and are displayed using identical settings. Scale bar is 10 µm. **b**: Quantification of the calcium depletion experiments representatively shown in (a) by fluorescence intensity measurements. N= 20 – 40, n= 3. Statistical analysis was done with a two-tailed paired t-test, ns: not significant. $p > 0.05$. **c**: Quantification of the calcium depletion experiments representatively shown in (a) by MSRE-qPCR on the α-satellite locus, n= 4. Statistical analysis was done with a two-tailed unpaired t-test, ** $p < 0.01$. **d-f:** MCF10A-BiAD cells were analyzed 72 h post transfection with either control (siNT) or an E-cadherin targeting siRNA (siCDH1). **d:** Representative fluorescence microscopy images of sparse MCF10A-BiAD cells. E-cadherin staining was used to validate the siRNA knockdown. β-catenin staining was employed for cell shape recognition. Images were acquired and are displayed using identical settings. Scale bar is 10 µm. **e**: Quantification of E-cadherin knock-down experiments representatively shown in (d) by fluorescence intensity measurements. N= 20 – 60, n= 4. Statistical analysis was done with a two-tailed paired t-test, ns: not significant. $p > 0.05$. **f**: Quantification of E-cadherin knock-down experiments representatively shown in (d) by MSRE-qPCR on the α-satellite locus, n= 3. Statistical analysis was done with a two-tailed unpaired t-test, ns: not significant. $p > 0.05$. **g-h:** Sparsely seeded MCF10A-BiAD cells were treated with E-cadherin blocking antibody (5 µg/mL, 24 h). **g:** Representative fluorescence microscopy images of sparsely seeded MCF10A-BiAD cells treated with the E-cadherin blocking antibody. Shown are the mVenus reporter

signals and staining for E-cadherin. Images were acquired and are displayed using identical settings. Scale bar is 10 µm. **h**: Quantification of E-cadherin blocking experiments representatively shown in (g) by fluorescence intensity measurements. N= 30, n= 3. Statistical analysis was done with a two-tailed paired t-test, ns: not significant. $p > 0.05$. (b-c, e-f, h) In the dot blots, each dot is the mean value obtained for one biological repeat, the line indicates the mean of all biological repeats, and error bars represent their standard deviation. Paired measurements are indicated with the color coding.

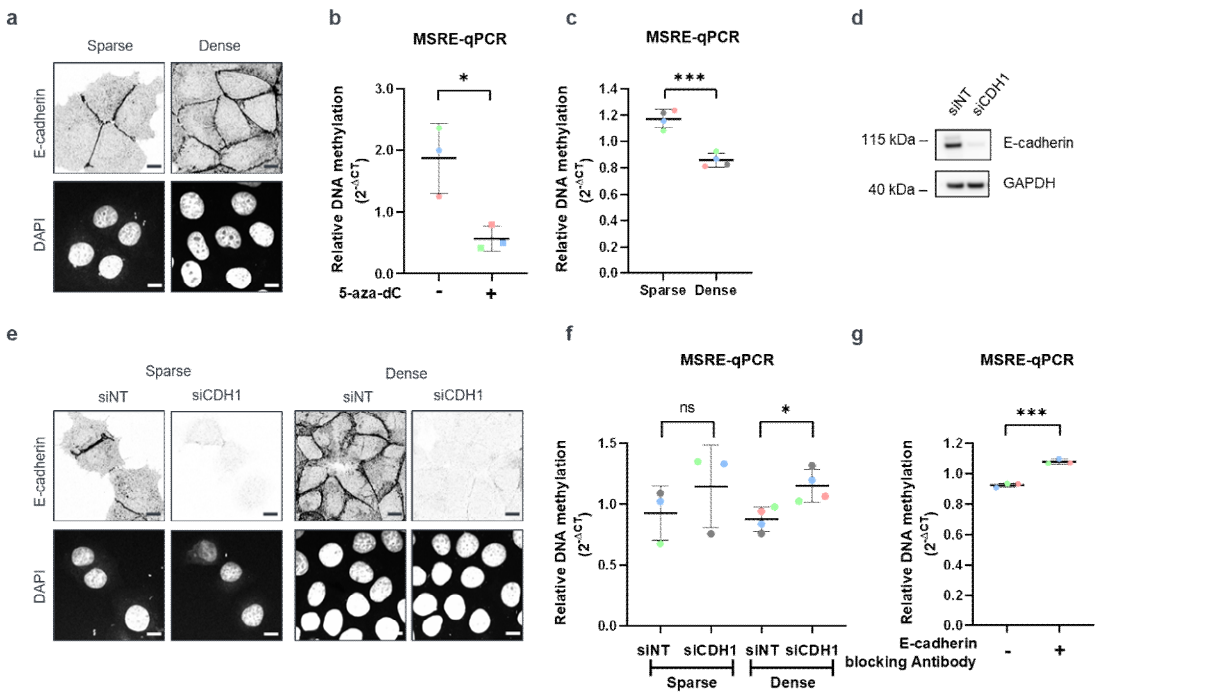

Figure S5: **The methylation status of α-satellite sequences in MCF7 cells is sensitive to cell confluency**. **a**: Representative immunofluorescence microscopy images showing E-cadherin expression and localization at adherens junctions in MCF7 cells. Nuclei were counterstained with DAPI. Images were acquired and are displayed using identical settings. Scale bar is 10 µm. **b**: MCF7 cells were treated for 24 h with 2 µM 5-aza-dC followed by genomic DNA isolation and MSRE-qPCR of the α-satellite locus. Mock treated cells were used as a control. Statistical analysis was done with a two-tailed unpaired t-test, $*p < 0.05$. **c**: MCF7 cells were cultured for 24 h under either sparse or dense conditions followed by genomic DNA isolation and MSRE-qPCR of the α-satellite locus. n= 4. Statistical analysis was done with a two-tailed unpaired t-test, $***p < 0.001$. **d-f**: MCF7 cells were analyzed 72 h post transfection with either control (siNT) or an E-cadherin targeting siRNA (siCDH1). Efficient knock-down of E-cadherin is shown by Western blot (**d**) as well as by representative fluorescence microscopy images (**e**). Images were acquired and are displayed using identical settings. Scale bar is 10 µm. For the Western blots shown in (d), antibody probing was performed on the same membrane strip, which was cut horizontally accordingly to the expected molecular weight of the target proteins. See also Figure S8b for the uncropped Western blot membranes. **f**: MCF7 cells were transfected with either control (siNT) or an E-cadherin targeting siRNA (siCDH1). 48h after knock-down, the cells were re-seeded under either sparse or dense conditions. Genomic DNA was isolated 24 h later and used for MSRE-qPCR analysis of the α-satellite DNA locus. n= 3-4. Statistical analysis was done with a two-tailed unpaired t-test, ns: not significant, $*p < 0.05$. **g**: MCF7 cells were treated with an E-cadherin blocking antibody, seeded under dense conditions and the methylation levels of α-satellites were assessed 24 h later by MSRE-qPCR. n= 3. Statistical analysis was done with a two-tailed unpaired t-test, $***p < 0.001$. (b-c, f-g) In the dot blots, each dot is the mean value obtained for one biological repeat, the line indicates the mean of all biological repeats, and error bars represent their standard deviation. Paired measurements are indicated with the color coding.

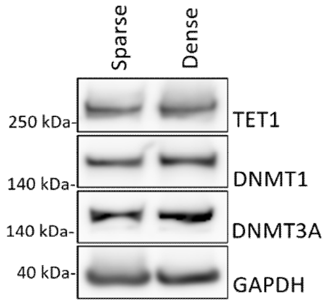

Figure S6: Western blot analysis of DNMT and TET1 enzymes in cell lysates obtained from sparse and confluent MCF10A cultures. Lysates were prepared 24 h after cell seeding and subjected to SDS-PAGE followed by Western blot analysis with the indicated antibodies. GAPDH was used as loading control. For antibody probing, the same full cell lysate was loaded 4 times in neighbouring lanes of the same SDS-PAGE gel. Each Western blot membrane strip was probed with one antibody (see also Figure S8f).

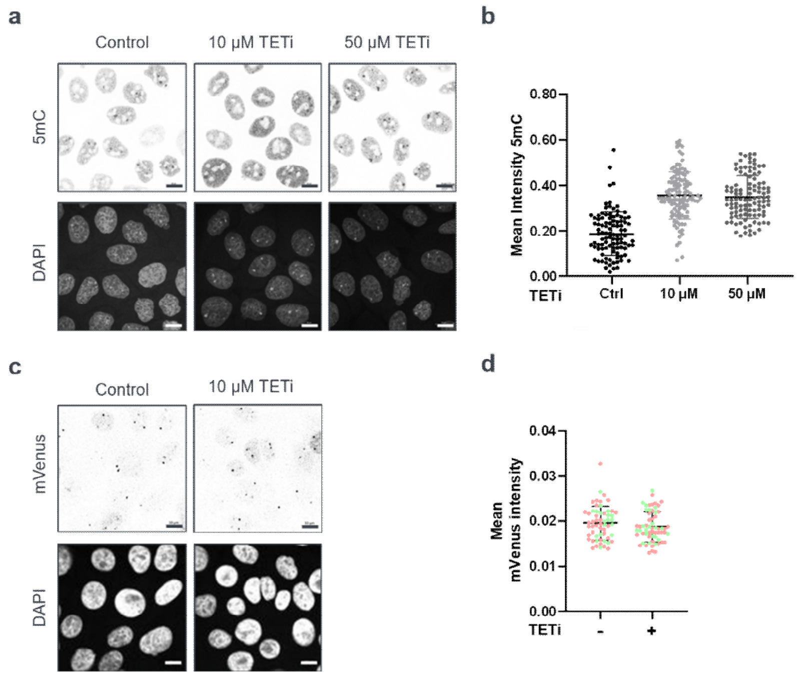

Figure S7: TET inhibition does not alter the methylation status of repeats in confluent BiAD cells. a-b Establishment of the Bobcat339 treatment conditions. MCF10A cells were seeded on collagen coated cover slips followed by treatment with different concentrations of Bobcat339. Mock treated cells served as control. The efficiency of TET inhibition was monitored by global 5mC staining. Nuclei were counterstained with DAPI. **a:** Shown are representative confocal microscopy images, which were acquired and are displayed under identical conditions. Scale bar is 10 µm. **b:** Quantification of the immunofluorescent experiments representatively shown in (a). Each dot is one cell. **c-d:** MCF10A-BiAD cells were seeded under dense conditions and treated with 10 µM Bobcat339 for 24 h before fixation and mVenus signal analysis. Nuclei were counterstained with DAPI. Mock treated cells were used as a control. **c:** Shown are representative confocal microscopy images, which were acquired and are displayed under identical conditions. Scale bar is 10 µm. **d:** Quantification of the immunofluorescent experiments representatively shown in (a). Each dot is one cell. Colors denote the biological repeat number used to collect the single cell data from (n=2).

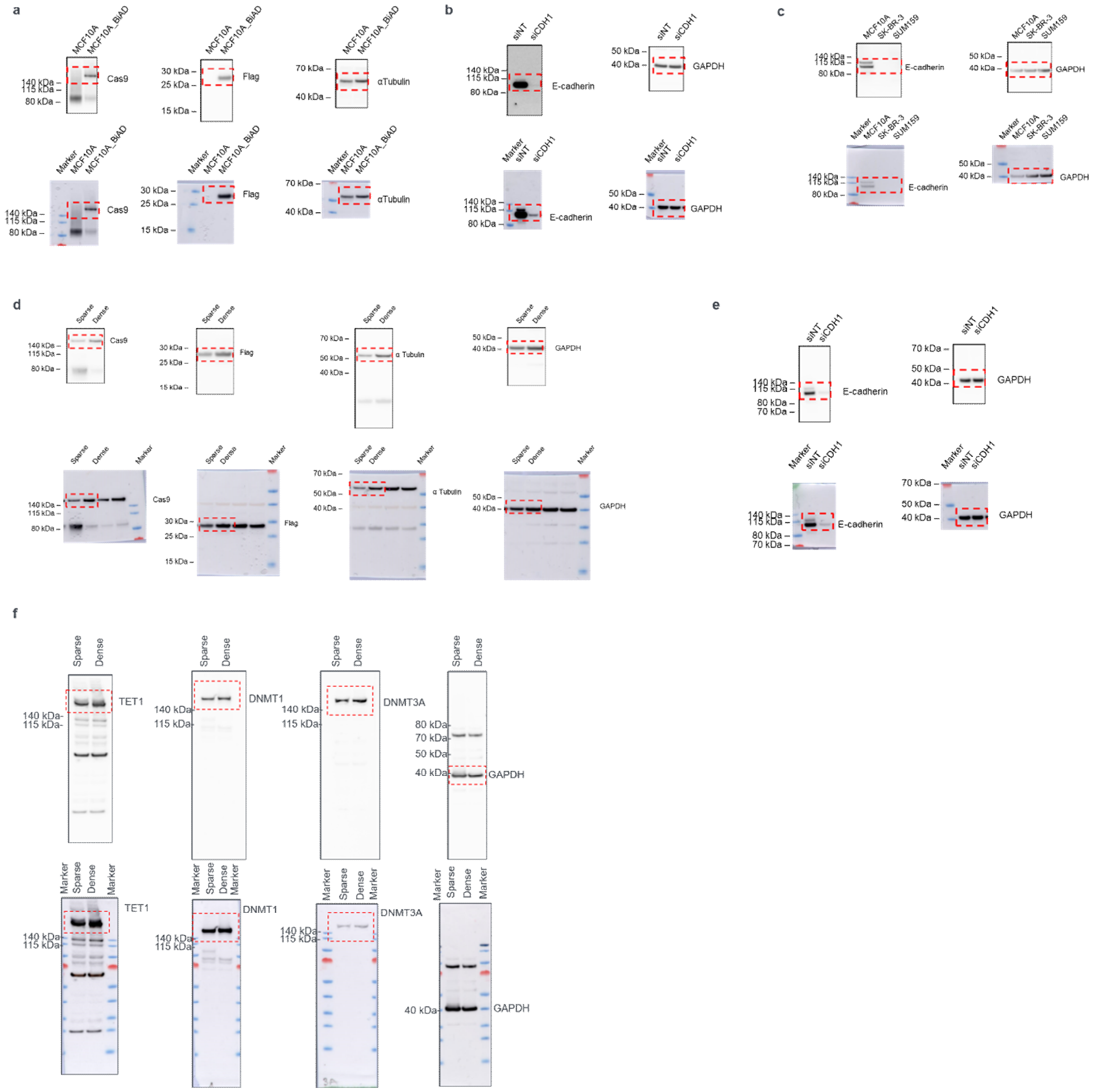

Figure S8: Uncropped Western blots. For each panel, the top row shows the chemiluminescence image, which was used to generate the figures in the manuscript. The bottom row shows the corresponding overlay with the calorimetric marker. **a:** Uncropped Western blot of images shown in Fig. 1b. **b:** Uncropped Western blot of images shown in Fig. 3d. **c:** Uncropped Western blot of images shown in Fig. 4g. **d:** Uncropped Western blot of images shown in Fig. S1b. **e:** Uncropped Western blot of images shown in Fig. S5d. **f:** Uncropped Western blot of images shown in Fig. S6.

Table S1: Summary of p values and sample sizes

Figure		Samples	n	p value	p value summary
**Figure 1**	d	Untreated vs Aza treated Microscopy	5	0.0383	*
	e	Untreated vs Aza treated MSRE-qPCR	3	0.0145	*
**Figure 2**	b	Sparse vs. dense Microscopy	4	0.0238	*
	c	Sparse vs. Dense - Area	5	0.7706	ns
	e	Sparse vs. Dense MFI	5	0.0003	***
	f	Sparse vs. Dense MSRE-qPCR	3	0.0031	**
**Figure 3**	b	Untreated vs Calcium Depl. Microscopy	4	0.0154	*
	c	Untreated vs Calcium Depl. MSRE-qPCR	4	<0.0001	****
	f	Sparse vs. Dense Microscopy	4	0.0097	**
		Dense vs. E-cadherin KD	4	0.0183	*
		Sparse vs. E-cadherin KD	4	0.9701	ns
	g	siNT vs. siCDH1 MSRE-qPCR	3	0.0038	**
	i	Untreated vs E-cad blocking AB Microscopy	4	0.0075	**
	j	Untreated vs E-cad Blocking AB MSRE-qPCR	3	0.0027	**
	l	Untreated vs CytD Microscopy	4	0.0108	*
	m	Untreated vs CytD - MSRE-qPCR	3	0.0147	*
**Figure 4**	b	MSD Violin plot	1 (Sparse N=50; Dense N=140)	<0.0001	****
	c	MSD median dot plot	3	0.0359	*
	d	Untreated vs. Aza ncRNA	3	0.0056	**
	e	Sparse vs. Dense ncRNA	3	0.0027	**
	f	Untreated vs E-cad Blocking AB ncRNA	3	0.0028	**
	h	Sparse vs dense SKBR3 MSRE-qPCR	3	0.5340	ns
		Sparse vs dense SUM159 MSRE-qPCR	3	0.4004	ns
	i	Sparse vs dense ncRNA SKBR3	4	0.4129	ns
		Sparse vs dense ncRNA SUM159	4	0.7838	ns
**Figure S1**	c	Sparse vs. Dense – Cas9	3	0.6571	ns
		Sparse vs. Dense – Flag-MBD	3	0.5514	ns
**Figure S3**	b	Sparse vs. Dense G0/G1	3	0.2262	ns
		Sparse vs. Dense S	3	0.7431	ns
		Sparse vs. Dense G2/M	3	0.6564	ns
**Figure S4**	b	Untreated vs Calcium Depletion Microscopy	3	0.5238	ns
	c	Untreated vs Calcium Depletion MSRE-qPCR	4	0.0061	**
	e	siNT vs siCDH1 Microscopy	4	0.0894	ns
	f	siNT vs siCDH1 - MSRE-qPCR	4	0.6336	ns
	h	Untreated vs E-cad blocking AB Microscopy	3	0.1797	ns
**Figure S5**	b	Untreated vs Aza MSRE-qPCR	3	0.0196	*
	c	Sparse vs dense MSRE-qPCR	4	0.0003	***
	f	siNT vs siCDH1 – sparse	3	0.4036	ns
		siNT vs siCDH1 – dense	4	0.0173	*
	g	Untreated vs E-cad Blocking AB MSRE-qPCR	3	0.0003	***
