# Supplementary material for: Repeat DNA methylation is modulated by adherens junction signaling
Source: Commun Biol. 2024 Mar 7;7:286. doi: 10.1038/s42003-024-05990-4 (PMC10920906; doi:10.1038/s42003-024-05990-4)
Supplement: Supplementary file 2 — Description of Additional Supplementary Files [file 42003_2024_5990_MOESM2_ESM.pdf]

Description of Additional Supplementary Files

File name: Supplementary Data 1
Description: List with the first 1000 hits of the sgRNA blast, sorted by chromosome number.

File name: Supplementary Data 2
Description: The source data behind the graphs in the paper.
