# Supplementary material for: Repeat DNA methylation is modulated by adherens junction signaling
Source: Commun Biol. 2024 Mar 7;7:286. doi: 10.1038/s42003-024-05990-4 (PMC10920906; doi:10.1038/s42003-024-05990-4)
Supplement: Supplementary file 5 — Reporting Summary [file 42003_2024_5990_MOESM5_ESM.pdf]

nature portfolio

Corresponding author(s): Prof. Monilola Olayioye and Dr. Cristiana Lungu

Last updated by author(s): Feb 7, 2024

Reporting Summary

Nature Portfolio wishes to improve the reproducibility of the work that we publish. This form provides structure for consistency and transparency in reporting. For further information on Nature Portfolio policies, see our Editorial Policies and the Editorial Policy Checklist.

Statistics

For all statistical analyses, confirm that the following items are present in the figure legend, table legend, main text, or Methods section.

n/a	Confirmed	
☐	☒	The exact sample size (*n*) for each experimental group/condition, given as a discrete number and unit of measurement
☐	☒	A statement on whether measurements were taken from distinct samples or whether the same sample was measured repeatedly
☐	☒	The statistical test(s) used AND whether they are one- or two-sided<br>*Only common tests should be described solely by name; describe more complex techniques in the Methods section.*
☒	☐	A description of all covariates tested
☐	☒	A description of any assumptions or corrections, such as tests of normality and adjustment for multiple comparisons
☐	☒	A full description of the statistical parameters including central tendency (e.g. means) or other basic estimates (e.g. regression coefficient) AND variation (e.g. standard deviation) or associated estimates of uncertainty (e.g. confidence intervals)
☐	☒	For null hypothesis testing, the test statistic (e.g. *F*, *t*, *r*) with confidence intervals, effect sizes, degrees of freedom and *P* value noted<br>*Give P values as exact values whenever suitable.*
☒	☐	For Bayesian analysis, information on the choice of priors and Markov chain Monte Carlo settings
☒	☐	For hierarchical and complex designs, identification of the appropriate level for tests and full reporting of outcomes
☒	☐	Estimates of effect sizes (e.g. Cohen's *d*, Pearson's *r*), indicating how they were calculated

Our web collection on statistics for biologists contains articles on many of the points above.

Software and code

Policy information about availability of computer code

Data collection	Bio-Rad CFX Manager version 3.1, FlowJo v10.7.2, MACSQuant VYB, ImageQuant TL 8.1, Zeiss ZEN versions 2.3, Zeiss ZEN 3.3
Data analysis	Bio-Rad CFX Manager version 3.1, FlowJo v10.7.2, MATLAB, GraphPad Prism 8.0.2, ImageQuant TL 8.1; CellProfiler 4.2.5, Zeiss ZEN 2.3, Zeiss ZEN 3.3, Fiji, Phyton version 3.12.0.

For manuscripts utilizing custom algorithms or software that are central to the research but not yet described in published literature, software must be made available to editors and reviewers. We strongly encourage code deposition in a community repository (e.g. GitHub). See the Nature Portfolio guidelines for submitting code & software for further information.

Data

Policy information about availability of data

All manuscripts must include a data availability statement. This statement should provide the following information, where applicable:

- Accession codes, unique identifiers, or web links for publicly available datasets
- A description of any restrictions on data availability
- For clinical datasets or third party data, please ensure that the statement adheres to our policy

All data supporting the conclusions and findings included in this study are available within the article or Supplementary Information. Source data are provided with this paper. Any additional information required to reanalyze the data reported in this study is available from the corresponding authors upon request.

Research involving human participants, their data, or biological material

Policy information about studies with human participants or human data. See also policy information about sex, gender (identity/presentation), and sexual orientation and race, ethnicity and racism.

Reporting on sex and gender	n/a
Reporting on race, ethnicity, or other socially relevant groupings	n/a
Population characteristics	n/a
Recruitment	n/a
Ethics oversight	n/a

Note that full information on the approval of the study protocol must also be provided in the manuscript.

Field-specific reporting

Please select the one below that is the best fit for your research. If you are not sure, read the appropriate sections before making your selection.

☒ Life sciences ☐ Behavioural & social sciences ☐ Ecological, evolutionary & environmental sciences

For a reference copy of the document with all sections, see nature.com/documents/nr-reporting-summary-flat.pdf

Life sciences study design

All studies must disclose on these points even when the disclosure is negative.

Sample size	No sample size calculation was performed. Sample size was determined from similar experiments in the literature. For all experiments, a minimum of three biological replicates were analyzed per condition with the following exceptions: Figure S1A and S7D (two biological repeats) and Figure S7B (one biological repeat). These experiments were not used for statistical analysis.
Data exclusions	For the MSD experiments, outliers, defined as values with more than three scaled median absolute deviations from the median were removed before data plotting and statistical analysis. No data were excluded from analysis for the other experiments.
Replication	All attempts at replication of data contained in this manuscript were successful. The repeat (n) times are labeled in the figure legend and in supplemental Table S1.
Randomization	The work does not involve participant groups; therefore, randomization was not relevant to the study.
Blinding	The work does not involve participant groups; therefore, blinding was not relevant to the study.

Reporting for specific materials, systems and methods

We require information from authors about some types of materials, experimental systems and methods used in many studies. Here, indicate whether each material, system or method listed is relevant to your study. If you are not sure if a list item applies to your research, read the appropriate section before selecting a response.

Materials & experimental systems

n/a	Involved in the study	
☐	☒	Antibodies
☐	☒	Eukaryotic cell lines
☒	☐	Palaeontology and archaeology
☒	☐	Animals and other organisms
☒	☐	Clinical data
☒	☐	Dual use research of concern
☒	☐	Plants

Methods

n/a	Involved in the study	
☒	☐	ChIP-seq
☐	☒	Flow cytometry
☒	☐	MRI-based neuroimaging

Antibodies

Antibodies used	anti-β-Catenin (BD, # 610154, 1.0 mg/mL, IF 1:500), 5-methylcytosine (5-mC) (D3S2Z) (Cell Signalling, #28692S, IF 1:1600), anti-Cas9

Antibodies used	(Santa Cruz, sc-517386, 0.2 mg/mL, IF 1:100, WB 1:250), anti-DNMT3A (64B1446) (Novus Biologicals, NB120-13888SS, 1.0 mg/mL, WB 1:1000), anti-DNMT1 (abcam, ab188453, 1.1 mg/mL, WB 1:1000), anti-E-cadherin (Cell Signalling, #3195, 0.2 mg/mL, WB 1:1000, IF 1:200), anti-Flag M2 (Sigma Aldrich, F1804, 1.0 mg/mL, WB 1:1000), anti-DYKDDDDK Flag tag (D6W5B) (Cell Signalling, #14793, IF 1:100), anti-GAPDH (Sigma Aldrich, G9545, 1.0 mg/mL, WB 1:5000), anti-α-Tubulin (Millipore, #05-829, 1.0 mg/mL, WB 1:10000), anti-TET1 (E5F1O) (Cell Signaling, #40142, WB 1:1000).
Validation	Clone 14/Beta-Catenin: validated by supplier using beta-catenin positive cells; 5-methylcytosine (5-mC) validated by supplier using ELISA, dot blot, and MeDIP assays; : Cas9 7A9-3A3: validated in this study by using MCF10A cells that do not express the modules; anti-DNMT3A (64B1446) validated by vendor using DNM3A transfected cells; anti-DNMT1 (abcam, ab188453) validated by vendor on knockout cells; E-Cadherin (24E10): validated in this work by using siCDH1 knockdown cells; ANTI-FLAG® M2 (Sigma, F1804): validated in this work by using by using MCF10A cells that do not express the modules; DYKDDDDK Tag (D6W5B): validated by vendor by using untransfected cells; GAPDH: validated by vendor based on Western blot analysis; Tubulin clone DM1A: validated by vendor on A431 lysates; anti-TET1 (E5F1O) (Cell Signaling) validated by vendor using knockdown cells.

Eukaryotic cell lines

Policy information about cell lines and Sex and Gender in Research

Cell line source(s)	MCF10A (female) cells were provided by Prof. Andreas Hecht (University of Freiburg), MCF7 (female) cells were provided by Dr. Angelika Hausser (University of Stuttgart); SK-BR-3 (female) were obtained from CLS; SUM159 (female) were provided by Dr. Thordur Oskarsson, DKFZ; LentiX HEK293T (female) cells were provided by Dr. Philipp Rathert (University of Stuttgart);
Authentication	All cell lines were authenticated by STR profiling.
Mycoplasma contamination	All cell lines tested negative for mycoplasma contamination.
Commonly misidentified lines (See ICLAC register)	No commonly misidentified lines were used in this work.

Flow Cytometry

Plots

Confirm that:

☒ The axis labels state the marker and fluorochrome used (e.g. CD4-FITC).

☒ The axis scales are clearly visible. Include numbers along axes only for bottom left plot of group (a 'group' is an analysis of identical markers).

☒ All plots are contour plots with outliers or pseudocolor plots.

☒ A numerical value for number of cells or percentage (with statistics) is provided.

Methodology

Sample preparation	For mVenus signal quantification, MCF10A_BiAD cells were seeded as described in the manuscript under sparse or dense conditions. The cells were detached by trypsinization before flow cytometry measurements of the mVenus signal. Parental MCF10A cells, which did not contain the reporter, were used for gating. For cell sorting, the reporter cells were cultured sparsely and were treated as described above. The mVenus positive cells were isolated for further propagation in culture as either mixed populations or as single cell clones.<br>For cell cycle analysis, MCF10A cells were cultured and detached as above followed by staining with FxCycle Violet and analysis as described in the methods section. The fluorescence signal was acquired in the linear range.
Instrument	FACS Aria III (BD Bioscience) MACSQuant® VYB (Miltenyi Biotec)
Software	Data was collected by the BD FACSDiva software (Version 8.0.1) or MACSQuant VYB, post-aquisition analysis was performed with the FlowJo software (v10.7.2).
Cell population abundance	After infection with the BiAD modules and antibiotic selection, the mVenus positive population was isolated by flow cytometry. The positive cells accounted for circa 0.2% of the total population as defined on a stringent gating relative to the non fluorescent parental MCF10A cells. The purity of the sorted populations and of the single cell clone was confirmed by microscopy. For the cell cycle distribution, all FxCycle Violet positive cells, as determined based on the unstained control, were analyzed further. Positive cells accounted for circa 99% of the total population.
Gating strategy	The strategy used for gating is described in the histograms shown in Figure 2D and Figure S3A.

☐ Tick this box to confirm that a figure exemplifying the gating strategy is provided in the Supplementary Information.
